# Effects of Cerebellar Transcranial Magnetic Stimulation on the Motor Function of Patients With Stroke: A Systematic Review and Meta‐Analysis

**DOI:** 10.1002/brb3.70471

**Published:** 2025-04-18

**Authors:** Yongxin Zhu, Juncong Yang, Kun Wang, Xianwen Li, Jiahui Ling, Xie Wu, Lianhui Fu, Qi Qi

**Affiliations:** ^1^ Shanghai YangZhi Rehabilitation Hospital (Shanghai Sunshine Rehabilitation Center), School of Medicine Tongji University Shanghai China; ^2^ Shanghai University of Sport Shanghai China; ^3^ Kunshan Rehabilitation Hospital Kunshan Jiangsu China

**Keywords:** balance, cerebellum, limb motor function, stroke, transcranial magnetic stimulation

## Abstract

**Background:**

As the core of motor control and learning, the cerebellum is crucial for maintaining posture, regulating muscle tone, and coordinating movement. In recent years, there has been an increase in the number of studies on the application of cerebellar transcranial magnetic stimulation (cTMS) to motor dysfunction in patients with stroke. This review aims to analyze cTMS efficacy for stroke patients and further explore the specific effects of different stages of the disease, stimulation modes, stimulation intensity, and treatment duration.

**Methods:**

Six databases were searched comprehensively—CNKI, Wanfang, Web of Science, PubMed, The Cochrane Library, and Embase—to collect randomized controlled trials (RCTs) up to October 2024 that investigated the improvement of physical motor dysfunction in stroke patients using cTMS. Two researchers screened the literature, extracted data, and independently assessed the quality and risk of bias of the included studies using the PEDro scale and the Cochrane Risk of Bias Assessment Tool 2. Meta‐analysis was performed using RevMan 5.4.

**Results:**

A total of 20 RCTs with 812 participants were included. Meta‐analysis and sensitivity analysis revealed that cTMS significantly improved BBS (Random, MD = 5.19, 95%CI = 3.66–6.72, *p* < 0.00001), enhanced FMA‐LE scores (Random, MD = 1.88, 95%CI = 0.76–3.01, *p* = 0.001), shortened the TUG (Fix, MD = −1.64, 95%CI = −2.60 to −0.68, *p* = 0.0008), and 10MWT durations (Fix, MD = −7.66, 95%CI = −12.33 to −2.99, *p* = 0.001), and increased MEP amplitudes (Fix, MD = 0.45, 95%CI = 0.04–0.87, *p* = 0.03). Subgroup analysis of the BBS showed that cTMS had a significant effect on patients with stroke in the subacute phase (*p* < 0.00001), with improvements observed using HF‐rTMS (*p* < 0.0001), iTBS (*p* < 0.00001), and intensities ≤ 80%RMT (< 80% RMT, *p* < 0.0001; 80% RMT, *p* < 0.00001). cTMS consistently demonstrated superior effects compared to controls across different intervention durations (5–10 sessions, *p* = 0.009; 11–20 sessions, *p* < 0.00001; > 20 sessions, *p* < 0.00001).

**Conclusion:**

cTMS effectively improves motor function in patients with stroke, particularly during the subacute phase with excitatory stimulation and moderate intensities (≤ 80%RMT).

**Trial Registration:**

PROSPERO number: CRD42024540604

## Graphical abstract

cTMS can effectively improve the motor function of patients with stroke, with significant efficacy in balance and limb motor function. The therapeutic effect of cTMS was more pronounced when intervening in the subacute phase, and when the excitatory stimulation mode and moderate intensity (≤ 80% RMT) were used.

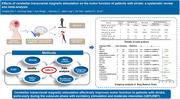

## 1 Introduction

According to global disease statistics, stroke is still the second leading cause of death in the world, and it is also the third leading cause of death and disease coexistence (GBD 2019 Stroke Collaborators 2021). According to relevant reports, the prevalence rate of stroke in China demonstrates a trend of stabilizing or continuous increase, with the annual number of newly reported first‐time stroke cases accounting for nearly one‐quarter of the total annual stroke cases globally. In particular, among individuals aged 40 and above, the prevalence rate soars to as high as 2.6% (Tu et al. 2023). More than 80% of patients with stroke suffer from motor dysfunction (Gorst et al. 2019; Xie et al. 2021), which leads to impaired balance, decreased mobility, and limited living ability, seriously affecting the quality of life of patients and their families. Although peripheral interventions such as traditional exercise therapy and neurodevelopmental therapy have demonstrated certain effectiveness in promoting functional recovery, given that the core problem of patients with stroke is the damage to the central nervous system, these peripheral interventions are relatively limited in promoting neuroplasticity, and thus their therapeutic effects have some limitations (Duncan et al. 2005).

Transcranial magnetic stimulation (TMS) is widely used in stroke rehabilitation and research as a non‐invasive, painless, and well‐tolerated brain stimulation technique (Hallett 2007; Fisicaro et al. 2019). Currently, the research and application of TMS primarily focus on the cerebral cortex. Among them, the primary motor cortex (M1) serves as the primary regulatory target for promoting motor and balance function recovery, and the reorganization of its neural function is crucial for the rehabilitation process (Kang et al. 2020). Despite the extensive clinical application research on the cerebral cortex as a core regulatory area, current rTMS treatment guidelines still fail to provide recommendations for cortical targets based on clear evidence (Lefaucheur et al. 2020). Given the complexity of the pathological mechanisms of stroke, its potential impact on a wide range of brain networks cannot be ignored (Ward 2004). As a key node capable of regulating multiple neural circuits, the cerebellum has gradually received attention and become an emerging target for brain function regulation (Bostan et al. 2010; Bostan et al. 2013). Since Koch et al. found the significant efficacy of cerebellar intermittent theta‐burst stimulation (iTBS) in improving walking and balance in patients with chronic stroke, this target has attracted extensive attention from the academic community (Koch et al. 2019). Furthermore, a study has found that stimulating the cerebellum to improve balance is superior to stimulating M1 (Liao et al. 2024). As the core of motor control and learning, the cerebellum is crucial for maintaining posture, regulating muscle tone, and coordinating movement. Cerebellar TMS (cTMS) not only regulates the cerebellar cortex but also influences the distant M1 and related functional areas (Harrington and Hammond‐Tooke 2015).

Although cTMS has been used in the rehabilitation of patients with motor dysfunction, controversy remains regarding its exact clinical efficacy. Meta‐analyses have shown that cTMS significantly improves balance and walking function in patients with stroke (Wu et al. 2022; Zeng et al. 2024; J. Wang et al. 2024). However, some studies have also found that the improvement in the Berg Balance Scale scores in the cerebellar hemisphere stimulation group did not reach the minimum clinically important difference compared to the control group (Liao et al. 2021; S. R. Wang and Li 2022). Furthermore, it has been found through imaging studies that this stimulation site coincides with the cognitive network nodes of the brain (O'Reilly et al. 2010). Studies acting on this target site have been found to significantly affect the functional connectivity of the cerebellum to the cognitive network of the brain, whereas there were no significant changes in the functional connectivity of the motor network (Rastogi et al. 2017).

In past meta‐analyses, Wu et al. (2022) synthesized relevant articles up to October 2021, while Zeng et al. (2024) and J. Wang et al. (2024) summarized the relevant research up to 2023. Over the past year, many new studies have been published (Zhu et al. 2024; Kong et al. 2024; Peng et al. 2024; Mao et al. 2024; Deng et al. 2024). However, the design and implementation of current clinical protocols still face challenges, lacking a unified and scientific framework for reference. Furthermore, the therapeutic effects of TMS are greatly influenced by both stimulation parameters and patients’ specific disease conditions, leading to significant heterogeneity in the results of the aforementioned meta‐analyses (Q. Liu et al. 2024). In light of this, it has become particularly urgent to conduct in‐depth explorations of key parameters such as different stimulation frequencies and intensities.

In view of this, this article intends to systematically review and integrate relevant research through meta‐analysis to evaluate the efficacy of cTMS on motor function in patients with stroke, and further explore the specific effects of different stages of disease, stimulation modes, stimulation intensity, and treatment duration, with a view to providing comprehensive and systematic guidance for rehabilitation therapy.

## 2 Methods

This study was conducted in accordance with the *Cochrane Handbook for Systematic Reviews 5.2.0* and was based on Preferred Reporting Items for the Systematic Reviews and Meta‐Analyses Statement 2020. This study has been registered on the PROSPERO International Systematic Review Registration Platform (https://www.crd.york.ac.uk/PRO‐SPERO) Registration No. CRD42024540604.

### 2.1 Search Strategy

The comprehensive search was conducted across CNKI, Wanfang Data, Web of Science, PubMed, The Cochrane Library, and Embase to collect randomized controlled trials (RCTs) on the improvement of motor function in patients with stroke through cTMS from the inception of these databases to October 2024. We used subject terms related to cTMS and stroke combined with free words. The following Mesh terms and keywords were used: “Stroke,” “Cerebrovascular Accident,” “CVA,” “Cerebrovascular Apoplexy,” “Brain Vascular Accident,” “Cerebrovascular Stroke,” “Transcranial Magnetic Stimulation,” “transcranial magnetic stimulat*,” “TMS,” “repetitive transcranial magnetic stimulat*,” “repetitive transcranial magnetic stimulation,” “rTMS,” “theta burst stimulation,” “theta burst stimulat*,” “TBS,” “stimulation,” “stimulat*,” “Cerebellum,” “Corpus Cerebelli,” “Cerebella,” “Parencephalon,” “cerebellar.” The detailed search strategy refers to the Appendix.

### 2.2 Inclusion and Exclusion Criteria

We conducted this analysis based on the PICOS (Population, Interventions, Comparators, Outcomes, and Study Design) framework for literature selection.

#### 2.2.1 Inclusion Criteria

- Population: The research subjects meet the clinical diagnostic criteria for stroke or are diagnosed with stroke by MRI or CT, encompassing both cerebral hemorrhage and cerebral ischemia;
- Interventions: The intervention measures were that the experimental group received cTMS, while receiving conventional rehabilitation therapy;
- Comparators: The control group received sham stimulation and received conventional rehabilitation therapy;
- Outcomes: The outcomes include at least one of the following: Berg Balance Scale (BBS), Fugl‐Meyer Assessment of Upper Extremity (FMA‐UE), Fugl‐Meyer Assessment of Lower Extremity (FMA‐LE), 10‐Metre Walking Test (10MWT), Timed Up and Go Test (TUG), Motor‐Evoked Potential (MEP);
- Study Design: RCTs;
- Publication Period and Country: There is no restriction on the publication period and country.

#### 2.2.2 Exclusion Criteria

- Population: Subjects with neurological diseases other than stroke, such as multiple sclerosis, essential tremor, intracranial infection, and brain trauma, were excluded;
- Interventions: Studies involving non‐invasive brain stimulation methods other than TMS, such as transcranial electrical stimulation, were excluded; Studies exploring the combined efficacy of specific medications with cTMS, in addition to conventional rehabilitation therapy, were excluded.
- Data Availability: Unable to obtain complete original data and no results within one month after contacting the author;
- Study Design: Non‐randomized, cross‐over study design and non‐controlled trials, conference papers, case reports, reviews, animal experiments, abstracts and reviews, and repeated publications.

### 2.3 Study Selection and Data Extraction

During the process of literature screening and evaluation, to ensure the objectivity and accuracy of the results, we adopted a double‐blind independent screening strategy. Two researchers (Y.Z. and J.Y.) independently conducted preliminary screening based on the titles and abstracts of the literature. Subsequently, each researcher thoroughly read the full text of the remaining articles, strictly adhered to the preset inclusion and exclusion criteria, independently assessed the applicability of each article, and extracted key data for preliminary evaluation. The two researchers cross‐checked their respective screening results. In cases of disagreement or inconsistency, they would discuss or consult the third researcher (K.W.) to jointly review and make a final decision.

Data extraction encompassed the following: (a) basic information of the literature (first author's name, publication year, sample size); (b) characteristics of the subjects (age and duration of illness); (c) intervention details (stimulation target, mode, intensity, number of pulses, duration of treatment); (d) evaluation indicators; (e) adverse reactions; (f) TMS devices.

### 2.4 Outcomes

We investigated the effects of cTMS on the balance and limb function of patients with stroke. The primary outcome was the BBS, and the secondary outcomes included the TUG, the FMA‐UE, the FMA‐LE, the 10MWT, and the MEP.

#### 2.4.1 The Primary Outcome

**BBS**: The BBS is the most widely used scale for evaluating the balance performance of patients with neurological diseases. It has good interrater and intrarater reliability. The total score is 56 points, consisting of 14 items, with each item scored from 0 to 4 points. A higher score indicates better balance ability. It includes multiple balance‐related movements such as sitting‐to‐standing, standing without support, standing with eyes closed, single‐leg standing, turning 360 degrees, alternating stepping on steps with both feet, and sitting down from a standing position (Meseguer‐Henarejos et al. 2019).

#### 2.4.2 Secondary Outcomes

**TUG**: The TUG records the time in seconds. The shorter the time, the better the postural control ability. The subject is required to stand up from a chair, walk forward 3 m at their normal walking gait, then turn around and come back to sit down. The time taken for the whole process is recorded. The shorter the time used, the better the postural control ability (Hafsteinsdóttir et al. 2014).

**FMA‐UE**: The Fugl‐Meyer Assessment is a recognized method for evaluating the motor function of patients with stroke. Among them, the FMA‐UE assesses the reflex activity, motor control, and muscle strength of the upper limb in hemiplegic patients after stroke. The FMA‐UE includes 33 items, and each item is scored on a scale of 0 to 2, where 0 = unable to perform, 1 = partially performed, and 2 = fully performed. It includes tests of various upper‐limb motor functions, such as the movement of the wrist, fingers, shoulders, and elbows (Singer and Garcia‐Vega 2017).

**FMA‐LE**: This assessment has good reliability in evaluating the lower‐limb motor control of patients with stroke. It includes a total of 17 assessment items for the lower limb. The FMA is an ordinal scale, and each item has three scores. If the subject cannot complete a certain task, they get 0 points; if they complete it partially, they get 1 point; and if they complete it fully, they get 2 points. For reflex activities, there are only two scores, 2 points for presence and 0 points for absence. The full score is 34 points, and a higher score indicates better lower‐limb motor function (Xie et al. 2021).

**10MWT**: The 10MWT requires the subject to complete a 10‐m timed walk at a comfortable speed on a 14‐m‐long straight‐line horizontal sidewalk, with 2 m at the front and back for acceleration and deceleration, respectively. The test needs to be repeated three times, and the final result is reported as the walking speed (m/s) converted from the average time (seconds) taken to walk 10 m, which effectively reflects the patient's walking ability (Van Criekinge et al. 2024).

**MEP**: As a direct marker of corticospinal tract excitability, MEP provides an important means for neurophysiological examination by stimulating the cerebral cortex and recording the responses of the spinal cord and muscles. Usually, TMS is used to activate the primary motor cortex, and the resulting action potential is recorded on the corresponding muscles, providing an objective basis for evaluating the integrity of neural pathways and functional recovery (Rosso et al. 2022).

### 2.5 Quality of Evidence

According to the *Cochrane Handbook for Systematic Reviews of Interventions version 5.2.0* (https://www.cochrane.org/), two researchers (Y.Z. and J.Y.) independently assessed the quality of all included studies using the Physiotherapy Evidence Database (PEDro) scale. For any discrepancies, the third researcher (K.W.) was consulted. The PEDro scale includes 11 items such as random allocation, blinding, dropout rate, and statistical reporting. The composite score ranges from 0 to 10, with higher scores indicating higher quality. The methodological quality was categorized as high (Hallett 2007; Fisicaro et al. 2019; Kang et al. 2020; Lefaucheur et al. 2020; Ward 2004), moderate (Xie et al. 2021; Duncan et al. 2005), and low (≤3). In addition, based on the GRADE rating scale and using the scale generated by GRADEpro GDT (https://gdt.gradepro.org/app/), we evaluated the quality of evidence in terms of risk of bias, inconsistency, indirectness, imprecision, and publication bias. We assessed the quality of evidence for primary and secondary outcomes and rated them as high, moderate, low, or very low. (a) High: we are very confident that the true effect is close to the estimated value of the effect. (b) Moderate: we have moderate confidence in the effect estimate. The true effect is likely to be close to the estimated value of the effect, but it may also differ substantially. (c) Low: our confidence in the effect estimate is limited. The true effect may differ greatly from the estimated value of the effect. (d) Very low: our confidence in the effect estimate is extremely low. The true effect is likely to differ significantly from the estimated value of the effect (Balshem et al. 2011).

### 2.6 Risk of Bias Assessment

Two researchers independently conducted bias risk assessments for all included articles using the Cochrane Risk of Bias 2.0 (RoB2) tool. The assessments covered five domains: “Randomization process,” “Deviations from intended interventions,” “Missing outcome data,” “Measurement of the outcome,” and “Selection of the reported result”. The bias risk was categorized as low risk, some concerns, and high risk. In cases of disagreement, the researchers resolved it through discussion or by consulting a third researcher.

### 2.7 Data Analysis

Statistical analysis was performed using RevMan 5.4 to extract the post‐intervention and follow‐up data of the test and control groups and calculate the mean difference (MD) and confidence interval (95% confidence interval, 95% CI). Heterogeneity was assessed using the *I* ^2^ test. If *p* > 0.05 and *I* ^2^ < 50%, indicating high homogeneity, a fixed‐effects model was employed; if *p* ≤ 0.05 and *I* ^2^ ≥ 50%, indicating significant heterogeneity, a random‐effects model was used. Subgroup analyses were conducted for BBS based on different ages (≤ 60 years vs. > 60 years) (Li et al. 2018), stroke phases (subacute vs. chronic), treatment modes(HF‐rTMS vs. LF‐rTMS vs. iTBS), intensities (< 80%RMT vs. 80%RMT vs, > 80%RMT), and total interventional sessions (5–10 sessions vs. 11–20 sessions vs. >20 sessions) with Bonferroni correction (α = 0.05/5) applied, where *p* < 0.01 was considered significant to reduce the risk of Type I error. Sensitivity analysis was performed using the one‐by‐one exclusion method. If data could not be combined for analysis, descriptive analysis was conducted instead.

## 3 Results

### 3.1 Trial Selection

**FIGURE 1:**
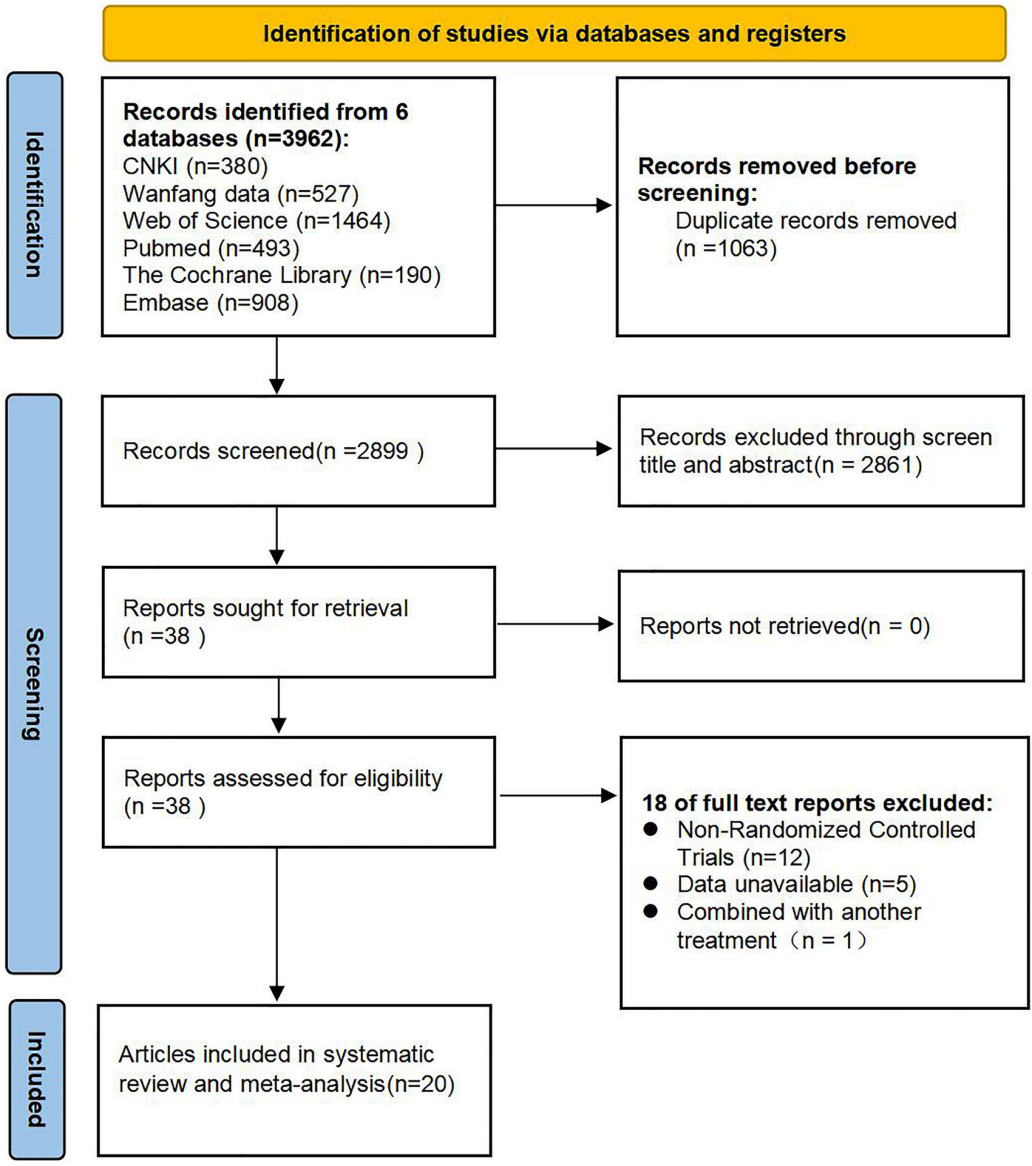
The PRISMA flowchart for study selection.

We retrieved a total of 3962 records from the databases of Web of Science (*n* = 1464), PubMed (*n* = 493), The Cochrane Library (*n* = 190), Embase (*n* = 908), CNKI (*n* = 380), and Wanfang Data (*n* = 527). After removing duplicate records (*n* = 1,063), we screened the titles and abstracts of the remaining 2,899 records and identified 38 records that seemed to meet the selection criteria. Upon reading the full texts, 20 studies were ultimately included. The PRISMA flowchart for study selection is depicted in Figure 1.

### 3.2 Study Characteristics

A total of 20 RCTs with a total of 812 subjects were included. The TMS devices utilized in these studies comprised the CCY series, CCI‐II (manufactured by YIRUIDE medical, Wuhan, China) (Xie et al. 2021; Liao et al. 2021; S. R. Wang and Li 2022; Zhu et al. 2024; Kong et al. 2024; Deng et al. 2024; Hu et al. 2017; Duan et al. 2020; Y. Chen et al. 2021; Mao et al. 2021; J. Chen et al. 2023; J. J. Zhang and Shi 2019; Ding et al. 2022), MagPro (produced by Medtronic, Minneapolis, MN, USA; and Magstim, Wales, UK) (Kim et al. 2014; Cha 2017), Magstim Rapid magnetic biphasic stimulator (Magstim Company) (Koch et al. 2019), Magpro R30 (Magventure, Farum, Denmark) (Im et al. 2022), Magstim 200 (MAGSTIM, UK) (Shan et al. 2022), and OSF‐6 (manufactured by Aosaifu Medical, Wuhan, China) (Mao et al. 2024). Predominantly, the figure‐8 coil was utilized in these studies. Of these, 16 studies reported BBS scores (Koch et al. 2019; Liao et al. 2021; S. R. Wang and Li 2022; Zhu et al. 2024; Kong et al. 2024; Peng et al. 2024; Mao et al. 2024; Deng et al. 2024; Hu et al. 2017; Mao et al. 2021; J. Chen et al. 2023; J. J. Zhang and Shi 2019; Ding et al. 2022; Kim et al. 2014; Im et al. 2022; Shan et al. 2022), 9 studies reported FMA‐LE scores (Xie et al. 2021; S. R. Wang and Li 2022; Zhu et al. 2024; Kong et al. 2024; Peng et al. 2024; Deng et al. 2024; Duan et al. 2020; Mao et al. 2021; J. Chen et al. 2023), 1 study reported FMA‐UE scores (Mao et al. 2021), 4 studies reported 10MWT results (Xie et al. 2021; J. Chen et al. 2023; Kim et al. 2014; Im et al. 2022), 5 studies reported TUG results (Xie et al. 2021; Zhu et al. 2024; Ding et al. 2022; Cha 2017; Im et al. 2022), and 4 studies reported MEP results (Liao et al. 2021; Kong et al. 2024; Duan et al. 2020; Y. Chen et al. 2021). The characteristics of the included studies are presented in Table 1.

**TABLE 1:** Characteristics of the included studies.

Authors, year (reference)	*n* (T/C)	Mean age (M±SD) Year		Mean duration (M±SD)		Stimulation target	Treatment parameters	Outcomes	Adverse events	TMS devices
		T	C	T	C					
Kim et al., 2014 (Kim et al. 2014)	22/10	67.4±7.8	64.8±11.7	16.8±13.4 days	14.0±4.9 days	The cerebellar hemisphere ipsilateral to the ataxic side: 2 cm below the inion and 2 cm lateral to the midline	rTMS(1 Hz) 100%RMT 900 pulses per session 15 min per session 1 session per day 5 days	10MWT BBS	No adverse events	Figure‐8 coil MagPro (Medtronic, Minneapolis, MN, USA)
Hu et al., 2017 (Hu et al. 2017)	20/20	56.8	57.3	48–72 h	48–72 h	The contralateral cerebellum	rTMS(5 Hz) 80%MT 100 pulses per session 20 min per session 7 sessions per week 4 weeks	BBS TGBS	/	Double coil B9076 CCY‐II magnetic stimulator (YIRUIDE medical, Wuhan, China)
Cha 2017 (Cha 2017)	15/15	61.60±7.76	63.73±6.10	75.20±12.91 days	77.20±10.02 days	The cerebellar hemisphere ipsilateral to the ataxic side: 2 cm below the inion and 2 cm lateral to the midline	rTMS(1 Hz) 100%RMT 900 pulses per session 15 min per session 5 sessions per week 4 weeks	WGS 6MWT TUG	No adverse events	Figure‐8 coi MagPro (Magstim, Wales, UK)
Zhang and Shi, 2019 (J. J. Zhang and Shi 2019)	15/15	54.33±11.46	55.53±13.13	5.20±3.60 weeks	5.47±2.58 weeks	The lateral cerebellum, contralateral to the affected hemisphere: 3 cm lateral to the midline and 1 cm below the inion	rTMS(10 Hz) 80%RMT 1200 pulses per session 20 min per session 5 sessions per week 2 weeks	BBS SI	/	CCY‐I magnetic stimulator (YIRUIDE medical, Wuhan, China)
Koch et al., 2019 (Koch et al. 2019)	17/17	63±11	65±12	≥6 months	≥6 months	The lateral cerebellum, contralateral to the affected hemisphere	iTBS 80%AMT 1200 pulses per session 10 min per session 5 sessions per week 3 weeks	BBS FMA BI TMS‐EEG	No adverse events	Figure‐8 coil Magstim Rapid magnetic biphasic stimulator (Magstim Company)
Duan et al., 2020 (Duan et al. 2020)	13/14	46.77±9.56	47.86±6.50	5.77±1.24 weeks	4.93±1.00 weeks	The contralateral cerebellum: 3 cm lateral to the midline and 1 cm below the inion	rTMS(1 Hz) 80%RMT 1600 pulses per session 1 session per day 4 weeks	FMA‐LE FMBS WGS RMT MEP	Mild pain in the side of the head was transiently treated	CCY‐I magnetic stimulator (YIRUIDE medical, Wuhan, China)
Chen et al., 2021 (Y. Chen et al. 2021)	16/16	57.38±8.04	51.44±9.19	80.13±35.19 days	101.50±54.15 days	The ipsilesional lateral cerebellum: 1 cm inferior to and 3 cm lateral to the inion	iTBS 80%AMT 600 pulses per session 5 sessions per week 2 weeks	MAS MTS SWV Hmax/Mmax MEP CMCT BI	No adverse events	Figure‐8 coil (YIRUIDE medical, Wuhan, China)
Xie et al., 2021 (Xie et al. 2021)	18/18	52.35±8.62	54.41±7.01	2.22±1.70 months	2.91±1.96 months	The contralesional cerebellum: 1 cm inferior to and 3 cm lateral to the inion	iTBS 80%AMT 600 pulses per session 3 min per session 1 session per day 10 consecutive weekdays	FMA‐LE 10MWT TUG FAC RMT MEP	No adverse events	Figure‐8 coil CCY‐I magnetic stimulator (YIRUIDE medical, Wuhan, China)
Liao et al., 2021 (Liao et al. 2021)	15/15	51.53±9.22	55.40±8.10	70.40±44.43 days	86.53±45.26 days	The cerebellar hemisphere contralateral to the affected cerebral hemisphere: 3 cm lateral to the midline and 1 cm below the inion	iTBS 80%AMT 600 pulses per session 5 sessions per week 2 weeks	BBS TIS FMA‐LE BI CSP RMT MEP	1 patient in the treatment group reported a mild headache	Figure‐8 coil CCY‐I rapid magnetic stimulator (YIRUIDE medical, Wuhan, China)
Mao et al., 2021 (Mao et al. 2021)	41/41	59.12±1.28	59.33±1.54	/	/	The lateral cerebellum, contralateral to the affected hemisphere: 3 cm lateral to the midline and 1 cm below the inion	rTMS(10 Hz) 110%RMT 1200 pulses per session 20 min per session 6 sessions per week 3 weeks	BBS BI FMA‐UE FMA‐LE SI	/	Figure‐8 coil CCY‐I rapid magnetic stimulator (YIRUIDE medical, Wuhan, China)
Im et al., 2022 (Im et al. 2022)	15/16	75.13±2.75	75.94±4.57	35.67±43.27 months	35.75±45.12 months	The cerebellar hemisphere contralateral to the site of cerebral infarction: 2 cm below and 2 cm lateral to the inion	rTMS(1 Hz) 90%RMT 900 pulses per session 15 min per session 5 sessions per week 2 weeks	BBS TUG 10MWT ABC	one subject complained of vertigo and discontinued treatment	Figure‐8 coil Magpro R30 (Magventure, Farum, Denmark)
Shan et al., 2022 (Shan et al. 2022)	30/30	59.25±10.89	59.25±10.96	3.02±0.61 months	2.79±0.53 months	The cerebellar hemisphere contralateral to the site of cerebral infarction	rTMS(1 Hz) 80% MT 100 pulses per session 20 min per session 6 sessions per week 4 weeks	BBS FM‐B RMT	/	Figure‐8 coil Magstim 200 (MAGSTIM, UK)
Wang and Li, 2022 (S. R. Wang and Li 2022)	21/21	52.62±8.61	54.62±7.85	82.33±45.27 weeks	72.95±47.37 weeks	The contralateral cerebellum: 3 cm lateral to the midline and 1 cm below the inion	iTBS 80%AMT 600 pulses per session 3 min per session 5 sessions per week 4 weeks	FMA‐LE BBS MBI MEP	No adverse events	Figure‐8 coil CCY‐I rapid magnetic stimulator (YIRUIDE medical, Wuhan, China)
Ding et al., 2022 (Ding et al. 2022)	38/38	56.65±9.54	56.31±9.14	5.43±1.54 weeks	5.37±1.78 weeks	The ipsilesional cerebellum: 3 cm lateral to the midline and 1 cm below the inion	rTMS(10 Hz) 80%RMT 1200 pulses per session 1 session per day 3 weeks	Gait analysis TUG SI BBS MRS	/	YRD CCI‐II (YIRUIDE medical, Wuhan, China)
Chen et al., 2023 (J. Chen et al. 2023)	16/16	58.88±15.79	62.38±12.66	3.75±2.84 months	3.88±2.53 months	The cerebellar hemisphere contralateral to the affected cerebral hemisphere:3 cm lateral to the midline and 1 cm below the inion	iTBS 80%RMT 600 pulses per session 200 seconds per session 6 sessions per week 3 weeks	BBS Ability to shift their center of gravity Total length of their shaking trajectory Maximum shaking diameter10MWT POMA‐G FMA‐LE BI	/	Figure‐8 coil CCY‐I rapid magnetic stimulator (YIRUIDE medical, Wuhan, China)
Mao et al., 2024 (Mao et al. 2024)	20/20	59.82±10.40	60.75±13.84	3.85±2.34 weeks	3.85±2.34 Weeks	The cerebellar hemisphere contralateral to the affected cerebral hemisphere:3 cm lateral to the midline and 1 cm below the inion	iTBS 600 pulses per session 2 sessions per day 10 sessions per week 2weeks	BBS FMA MBI FA ADC	No adverse events	Figure‐8 coil OSF‐6 (Aosaifu Medical,Wuhan,China)
Kong et al., 2024 (Kong et al. 2024)	15/15	59.60±8.85	62.87±8.91	2.17±1.68 months	1.83±1.56 months	The cerebellar hemisphere contralateral to the affected cerebral hemisphere:3 cm lateral to the midline and 1 cm below the inion	iTBS 80%RMT 600 pulses per session 1 session per day 5 sessions per week 3weeks	BBS FMA‐LE MBI FAC Gait analysis MEP	/	Circular coil CYY‐1 rapid magnetic stimulator (YIRUIDE medical, Wuhan, China)
Deng et al., 2024 (Deng et al. 2024)	25/25	52.64±13.04	48.28±16.77	4 (2,5) months	3 (1,9) months	The cerebellar hemisphere contralateral to the affected cerebral hemisphere:3 cm lateral to the midline and 1 cm below the inion	iTBS 80%RMT 600 pulses per session 6 sessions per week 3 weeks	BBS FMA‐LE BrunnstromMBI Gait analysis Isokinetic muscle strength testMEP	/	Figure‐8 coil CCY‐I rapid magnetic stimulator (YIRUIDE medical, Wuhan, China)
Peng et al., 2024 (Peng et al. 2024)	21/21	60.52±9.09	60.33±8.40	5.23±2.46 days	5.76±2.46 days	Cerebellar vermis: 1 cm below the inion	iTBS 70% 1200 pulses per session 1 session per day 6 sessions per week 2 weeks	BBS FMA‐LE 6MWT Isokinetic muscle strength testMAS MBI	No adverse events	Figure‐8 coil
Zhu et al., 2024 (Zhu et al. 2024)	18/18	58.67 ±7.24	62.33±8.78	61.89±46.72 days	52.00±48.56 days	The cerebellar hemisphere contralateral to the affected cerebral hemisphere:3 cm lateral to the midline and 1 cm below the inion	iTBS 80%AMT 600 pulses per session 2 sessions per day 5 sessions per week 2 weeks	BBS FMA‐LE TUG BI Gait analysis	No adverse events	Figure‐8 coil CCY‐I rapid magnetic stimulator (YIRUIDE medical, Wuhan, China)

Abbreviations: 10MWT, 10‐Meter Walk Test; 6MWT, 6‐Minute Walk Test; ABC, Activities‐Specific Balance Confidence Scale; ADC, Apparent Diffusion Coefficient; AMT, Active Motor Threshold; BBS, Berg Balance Scale; BI, Barthel Index; C, control group; CMCT, Central Motor Conduction Time; CSP, Cortical Silent Period; FA, Fractional Anisotropy; FAC, Functional Ambulation Categories; FMA, Fugl‐Meyer Assessment; FMA‐LE, Fugl‐Meyer Assessment—Lower Extremity; FMA‐UE, Fugl‐Meyer Assessment—Upper Extremity; FM‐B, Fugl‐Meyer Balance Scale; FMBS, Fugl‐Meyer Balance Scale; Hmax/Mmax, H‐max/M‐max Amplitude Ratio; iTBS, Intermittent Theta Burst Stimulation; MAS, Modified Ashworth Scale; MBI, Modified Barthel Index; MEP, Motor‐Evoked Potential; MRS, Magnetic Resonance Spectroscopy; MT, Motor Threshold; MTS, Modified Tardieu Scale; POMA‐G, Performance‐Oriented Mobility Assessment—Gait; RMT, Resting Motor Threshold; rTMS, Repetitive Transcranial Magnetic Stimulation; SI, Stability Index; SWV, Shear Wave Velocity; T, treatment group; TGBS, Tinetti Gait and Balance Scale; TIS, Trunk Impairment Scale; TMS‐EEG, Transcranial Magnetic Stimulation—Electroencephalography; TUG, Timed Up and Go Test; WGS, Wisconsin Gait Scale.

### 3.3 Quality of Evidence and Risk of Bias Assessment

**FIGURE 2:**
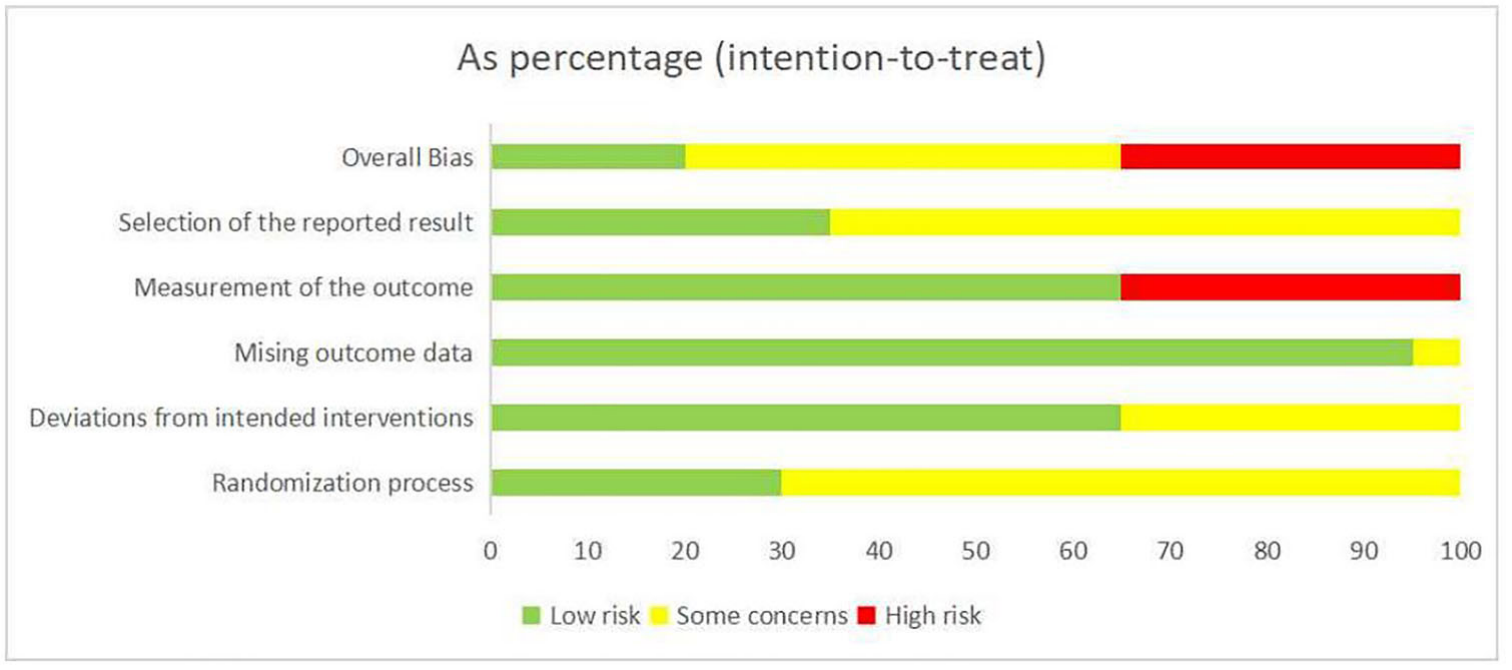
Risk of bias summary.

**FIGURE 3:**
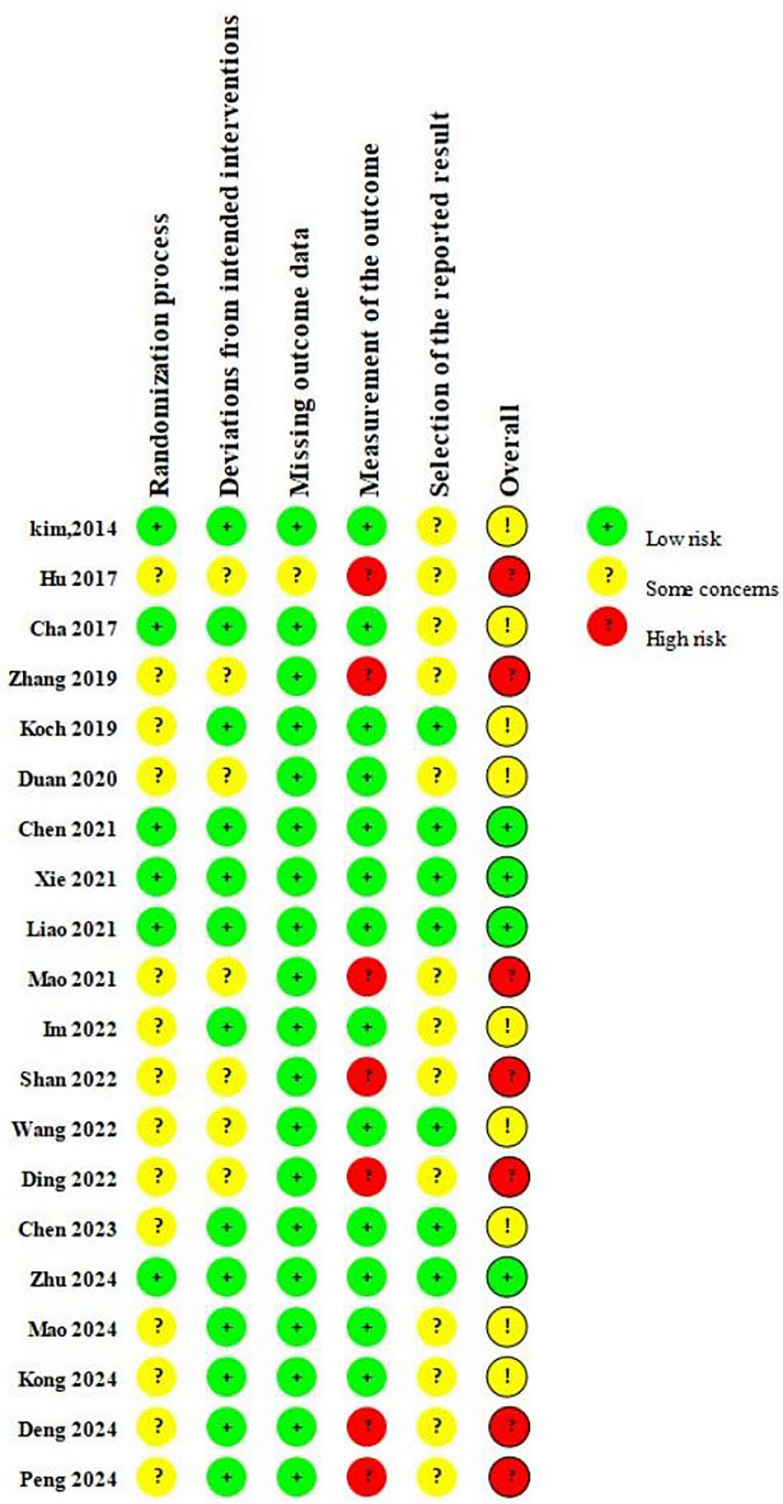
Risk of bias graph.

The overall scoring range of the PEDro scale is 6–9 points, with an average score of 7.6 points, indicating acceptable quality of the included studies. The PEDro scale is presented in Table 2. The risk of bias assessment is illustrated in Figures 2 and 3. All included studies mentioned randomization, with six studies providing detailed descriptions of methods for allocation concealment (Xie et al. 2021; Liao et al. 2021; Zhu et al. 2024; Y. Chen et al. 2021; Kim et al. 2014; Cha 2017), resulting in a low risk of bias in the randomization process. Seven studies failed to blind the outcome assessors (Peng et al. 2024; Deng et al. 2024; Hu et al. 2017; Mao et al. 2021; J. J. Zhang and Shi 2019; Ding et al. 2022; Shan et al. 2022), leading to a high risk of bias in outcome assessment. Additionally, one study had a dropout rate exceeding 15% (Cha 2017), resulting in incomplete outcome data. According to the evaluation of the GRADE system, considering the significant heterogeneity in BBS and FMA‐LE, which led to the determination of “serious” inconsistency, they were rated as moderate‐quality evidence. 10MWT, TUG, and MEP were rated as high‐quality evidence (Table 3).

**TABLE 2:** Methodological quality assessment of RCT's using PEDro scoring system.

Author, year (reference)	Eligibility	Randomized allocation	Concealed allocation	Baseline comparability	Blinded subject	Blinded therapists	Blinded raters	Key outcomes	Intention to treat	Comparison between groups	Precision and variability	Total (0–10)
Kim et al., 2014 (Kim et al. 2014)	√	√	√	√	√		√	√	√	√	√	9
Hu et al., 2017 (Hu et al. 2017)	√	√		√				√	√	√	√	6
Cha 2017 (Cha 2017)	√	√	√	√	√		√		√	√	√	8
Zhang and Shi, 2019 (J. J. Zhang and Shi 2019)	√	√		√				√	√	√	√	6
Koch et al., 2019 (Koch et al. 2019)	√	√		√	√		√	√	√	√	√	8
Duan et al., 2020 (Duan et al. 2020)	√	√		√			√	√	√	√	√	7
Chen et al., 2021 (Y. Chen et al. 2021)	√	√	√	√	√		√	√	√	√	√	9
Xie et al., 2021 (Xie et al. 2021)	√	√	√	√	√		√	√	√	√	√	9
Liao et al., 2021 (Liao et al. 2021)	√	√	√	√	√		√	√	√	√	√	9
Mao et al., 2021 (Mao et al. 2021)	√	√		√	√			√	√	√	√	7
Im et al., 2022 (Im et al. 2022)	√	√		√		√	√	√	√	√	√	8
Shan et al., 2022 (Shan et al. 2022)	√	√		√				√	√	√	√	6
Wang and Li, 2022 (S. R. Wang and Li 2022)	√	√		√			√	√	√	√	√	7
Ding et al., 2022 (Ding et al. 2022)	√	√		√	√			√	√	√	√	7
Chen et al., 2023 (J. Chen et al. 2023)	√	√		√	√		√	√	√	√	√	8
Mao et al., 2024 (Mao et al. 2024)	√	√		√	√		√	√	√	√	√	8
Kong et al., 2024 (Kong et al. 2024)	√	√		√	√		√	√	√	√	√	8
Deng et al., 2024 (Deng et al. 2024)	√	√		√	√			√	√	√	√	7
Peng et al., 2024 (Peng et al. 2024)	√	√		√				√	√	√	√	6
Zhu et al., 2024 (Zhu et al. 2024)	√	√	√	√	√		√	√	√	√	√	9

**TABLE 3:** GRADEpro GDT evidence quality.

Certainty assessment							No. of patients		Effect			
No. of studies	Study design	Risk of bias	Inconsistency	Indirectness	Imprecision	Other considerations	experimental	control	Relative (95% CI)	Absolute (95% CI)	Certainty	Importance
Berg Balance Scale												
16	Randomized trials	Not serious	Serious^a^	Not serious	Not serious	None	349	338	—	MD **5.19 higher** (3.66 higher to 6.72 higher)	⨁⨁⨁◯ Moderate^a^	Important
Fugl‐Meyer assessment—Lower Extremity												
9	Randomized trials	Not serious	Serious^a^	Not serious	Not serious	None	188	189	—	MD **1.88 higher** (0.76 higher to 3.01 higher)	⨁⨁⨁◯ Moderate^a^	Important
10‐Meter Walk Test												
4	Randomized trials	Not serious	Not serious	Not serious	Not serious	None	71	60	—	MD **7.66 lower** (12.33 lower to 2.99 lower)	⨁⨁⨁⨁ High	Important
Timed Up and Go Test												
5	Randomized trials	Not serious	Not serious	Not serious	Not serious	None	104	105	—	MD **1.64 lower** (2.6 lower to 0.68 lower)	⨁⨁⨁⨁ High	Important
Motor‐Evoked Potential												
4	Randomized trials	Not serious	Not serious	Not serious	Not serious	None	59	60	—	MD **0.45 higher** (0.04 higher to 0.87 higher)	⨁⨁⨁⨁ High	Important

^a^ *I* ^2^ = 76%.

### 3.4 Results of Meta‐Analysis

#### 3.4.1 Balance Function

**BBS**: In this analysis, 687 patients from 16 studies were included (Koch et al. 2019; Liao et al. 2021; S. R. Wang and Li 2022; Zhu et al. 2024; Kong et al. 2024; Peng et al. 2024; Mao et al. 2024; Deng et al. 2024; Hu et al. 2017; Mao et al. 2021; J. Chen et al. 2023; J. J. Zhang and Shi 2019; Ding et al. 2022; Kim et al. 2014; Im et al. 2022; Shan et al. 2022). Due to high heterogeneity (*I* ^2^ = 76%, *p* < 0.00001), a random‐effects model was used for analysis. The BBS scores in the experimental group were significantly higher than those in the control group (Random, MD = 5.19, 95% CI = 3.66–6.72, *p* < 0.00001). After sensitivity analysis, *I* ^2^was 41%, still showing consistent significance (*p* < 0.00001)(Table 4).

**TABLE 4:** Results of meta‐analysis.

Outcomes	Number of studies/*n*	Heterogeneity test		Effect model	Results of meta‐analysis	
		*I* ^2^	*p*		MD(95%CI)	*p*
BBS (Koch et al. 2019; Liao et al. 2021; S. R. Wang and Li 2022; Zhu et al. 2024; Kong et al. 2024; Peng et al. 2024; Mao et al. 2024; Deng et al. 2024; Hu et al. 2017; Mao et al. 2021; J. Chen et al. 2023; J. J. Zhang and Shi 2019; Ding et al. 2022; Kim et al. 2014; Im et al. 2022; Shan et al. 2022)	16	76%	< 0.00001	Random	5.19 (3.66, 6.72)	< 0.00001
TUG (Xie et al. 2021; Zhu et al. 2024; Ding et al. 2022; Cha 2017; Im et al. 2022)	5	27%	0.24	Fix	−1.64 (−2.60, ‐0.68)	0.0008
FMA‐LE (Xie et al. 2021; S. R. Wang and Li 2022; Zhu et al. 2024; Kong et al. 2024; Peng et al. 2024; Deng et al. 2024; Duan et al. 2020; Mao et al. 2021; J. Chen et al. 2023)	9	76%	< 0.0001	Random	1.88 (0.76, 3.01)	0.001
10MWT (Xie et al. 2021; J. Chen et al. 2023; Kim et al. 2014; Im et al. 2022)	4	5%	0.37	Fix	−7.66 (−12.33, −2.99)	0.001
MEP (Liao et al. 2021; Kong et al. 2024; Duan et al. 2020; Y. Chen et al. 2021)	4	10%	0.34	Fix	0.45 (0.04, 0.87)	0.03

Abbreviations: 10MWT, 10‐Meter Walk Test; BBS, Berg Balance Scale; TUG, Timed Up and Go Test; FMA‐LE, Fugl‐Meyer Assessment—Lower Extremity; MEP, Motor‐Evoked Potential.

Only four trials followed patients for what was considered to be long‐term follow‐up (Koch et al. 2019; J. Chen et al. 2023, Kim et al. 2014; Im et al. 2022). The follow‐up results showed that the BBS score of the experimental group was significantly higher than that of the control group (Random, MD = 5.46, 95% CI = 1.55–9.37, *p* = 0.006). However, due to significant heterogeneity (*I* ^2^ = 60%), after conducting a sensitivity analysis, the heterogeneity decreased to 43%, but the effect became statistically non‐significant (Random, MD = 3.23, 95% CI = −1.92 to 8.39, *p* = 0.22) (Table 5).

**TABLE 5:** Results of follow‐up.

Outcomes	Number of studies/*n*	Heterogeneity test		Effect model	Results of meta‐analysis	
		*I* ^2^	*P*		MD(95%CI)	*P*
BBS (Koch et al. 2019; J. Chen et al. 2023; Kim et al. 2014; Im et al. 2022)	4	60%	0.06	Random	5.46 (1.55, 9.37)	0.006
TUG (Im et al. 2022)	1	—	—	—	−2.30 (−12.65, 8.05)	0.66
FMA‐LE (J. Chen et al. 2023)	1	—	—	—	−0.82 (−2.09, 0.45)	0.21
10MWT (J. Chen et al. 2023; Kim et al. 2014; Im et al. 2022)	3	0%	0.47	Fix	−11.88 (−19.16, −4.60)	0.001

Abbreviations: 10MWT, 10‐Meter Walk Test; BBS, Berg Balance Scale; TUG, Timed Up and Go Test; FMA‐LE, Fugl‐Meyer Assessment—Lower Extremity.

**FIGURE 4:**
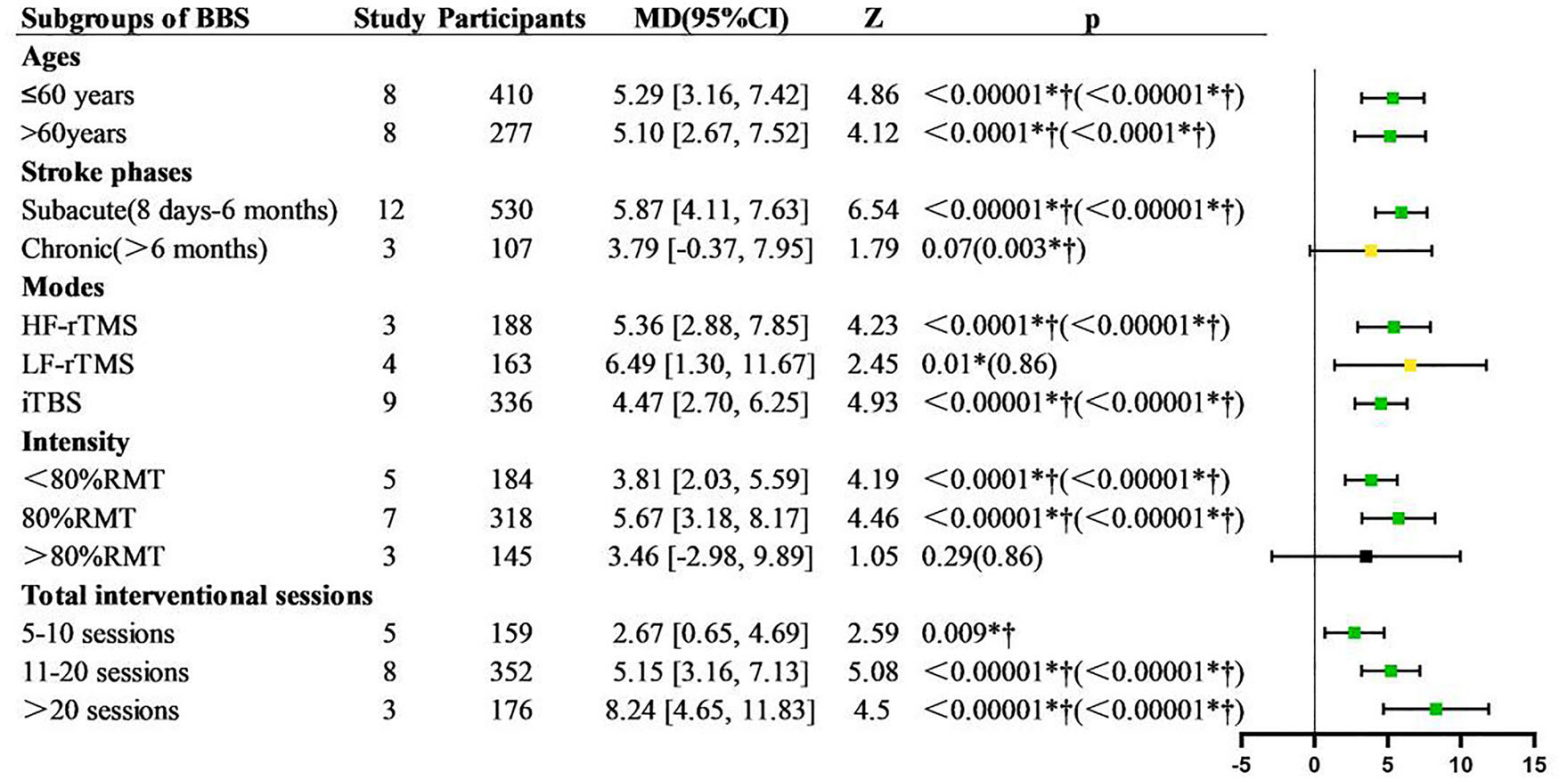
Subgroup analysis of BBS. Green 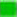 indicates “stable results with significant differences”; black 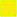 indicates “stable results without significant differences”; yellow 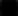 indicates “unstable results”; *p < 0.05; †Post hoc analysis *p* < 0.01. The value in () is the *p* value obtained after the sensitivity analysis. HF‐rTMS, high‐frequency repetitive transcranial magnetic stimulation; LF‐rTMS, low‐frequency repetitive transcranial magnetic stimulation; iTBS, intermittent theta burst stimulation; RMT, resting motor threshold.

Subgroup analysis results showed that in patients aged ≤ 60 years, the BBS scores in the experimental group were significantly higher than those in the control group (*p* < 0.00001), and this pattern was also observed in subjects older than 60 years (*p* < 0.0001). Among subacute patients (Liao et al. 2021; Zhu et al. 2024; Kong et al. 2024; Peng et al. 2024; Mao et al. 2024; Hu et al. 2017; Mao et al. 2021; J. Chen et al. 2023; J. J. Zhang and Shi 2019; Ding et al. 2022; Kim et al. 2014; Shan et al. 2022), the BBS scores in the experimental group were significantly higher than those in the control group, and this result remained stable under different analyses (*p* < 0.00001). For chronic patients (Koch et al. 2019; S. R. Wang and Li 2022; Im et al. 2022), although significant differences were observed after sensitivity analysis (*p* = 0.003), the initial comparison was not significant (*p* = 0.07). Among different stimulation modes, both high‐frequency repetitive TMS (HF‐rTMS) (Mao et al. 2021; J. J. Zhang and Shi 2019; Ding et al. 2022) and intermittent Theta‐burst stimulation (iTBS) (Koch et al. 2019; Liao et al. 2021; S. R. Wang and Li 2022; Zhu et al. 2024; Kong et al. 2024; Peng et al. 2024; Mao et al. 2024; Deng et al. 2024; J. Chen et al. 2023) significantly improved BBS scores (HF‐rTMS, *p* < 0.0001; iTBS, *p* < 0.00001), while low‐frequency repetitive TMS (LF‐rTMS) (Hu et al. 2017; Kim et al. 2014; Im et al. 2022; Shan et al. 2022) became insignificant after sensitivity analysis (*p* = 0.86). In terms of stimulation intensity, both 80% (Kong et al. 2024; Deng et al. 2024; Hu et al. 2017; J. Chen et al. 2023; J. J. Zhang and Shi 2019; Ding et al. 2022; Shan et al. 2022) and below 80% (Koch et al. 2019; Liao et al. 2021; S. R. Wang and Li 2022; Zhu et al. 2024; Peng et al. 2024) of resting motor threshold (RMT) intensity significantly improved BBS scores (< 80% RMT, *p* < 0.0001; 80% RMT, *p* < 0.00001), but no significant effect was observed when the intensity exceeded 80% RMT (Mao et al. 2021; Kim et al. 2014; Im et al. 2022). Additionally, regardless of short‐term (5–10 sessions) (Liao et al. 2021; Zhu et al. 2024; J. J. Zhang and Shi 2019; Kim et al. 2014; Im et al. 2022), medium‐term (11–20 sessions) (Koch et al. 2019; S. R. Wang and Li 2022; Kong et al. 2024; Peng et al. 2024; Mao et al. 2024; Deng et al. 2024; Mao et al. 2021; J. Chen et al. 2023), or long‐term treatment (> 20 sessions) (Hu et al. 2017; Ding et al. 2022; Shan et al. 2022), the BBS scores in the experimental group were significantly higher than those in the control group(Figure 4).

**TUG**: A total of 209 patients from five studies were included (Xie et al. 2021; Zhu et al. 2024; Ding et al. 2022; Cha 2017; Im et al. 2022). Due to the low heterogeneity (*I* ^2^ = 27%, *p* = 0.24), a fixed‐effects model was used for analysis. The TUG duration in the experimental group was significantly shorter than that in the control group (Fix, MD = −1.64, 95%CI = −2.60 to −.68, *p* = 0.0008) (Table 4).

One of the studies (Im et al. 2022) conducted a follow‐up on patients 4 weeks after the intervention. The follow‐up results indicated that the time taken for TUG test in the experimental group was not significantly shorter than that in the control group (MD = −2.30, 95% CI = −12.65 to 8.05, *p* = 0.66) (Table 5).

#### 3.4.2 Limb Motor Function

**FMA‐LE**: Data from 377 patients across 9 studies were included (Xie et al. 2021; S. R. Wang and Li 2022; Zhu et al. 2024; Kong et al. 2024; Peng et al. 2024; Deng et al. 2024; Duan et al. 2020; Mao et al. 2021; J. Chen et al. 2023). Due to high heterogeneity (*I* ^2^ = 76%, *p* < 0.0001), a random‐effects model analysis was used. The FMA‐LE scores in the experimental group were significantly higher than those in the control group (Random, MD = 1.88, 95%CI = 0.76–3.01, *p* = 0.001). After sensitivity analysis, *I* ^2^ was 31%, and the result remained significant (*p* < 0.00001) (Table 4).

One of the studies (J. Chen et al. 2023) followed up with patients 3 weeks after the intervention. The follow‐up results showed that the FMA‐LE scores of the experimental group were not significantly higher than those of the control group (MD = − 0.82, 95% CI = −2.09 to 0.45, *p* = 0.21) (Table 5).

**10MWT**: Data from 131 patients across 4 studies were included (Xie et al. 2021; J. Chen et al. 2023; Kim et al. 2014; Im et al. 2022). Because of the low heterogeneity, a fixed‐effects model analysis was used. The time taken to complete the 10MWT in the experimental group was significantly shorter than that in the control group (Fix, MD = −7.66, 95%CI = −12.33 to −2.99, *p* = 0.001) (Table 4).

Three of the studies (J. Chen et al. 2023; Kim et al. 2014; Im et al. 2022) followed up with patients 3 to 4 weeks after the intervention. The follow‐up results showed that the time taken for the 10MWT in the experimental group was significantly shorter than that in the control group (Fix, MD = −11.88, 95% CI = −19.16 to −4.60, *p* = 0.001) (Table 5).

#### 3.4.3 Cortical Excitability

**MEP**: Data from 119 patients across four studies were included (Liao et al. 2021; Kong et al. 2024; Duan et al. 2020; Y. Chen et al. 2021). Because of low heterogeneity (*I* ^2^ = 10%, *p* = 0.34), fixed‐effects model analysis was used. MEP was significantly higher in the trial group than in the control group (Fix, MD = 0.45, 95% CI = 0.04–0.87, *p* = 0.03) (Table 4).

#### 3.4.4 Safety

A total of 12 studies documented adverse events (Xie et al. 2021; Koch et al. 2019; Liao et al. 2021; S. R. Wang and Li 2022; Zhu et al. 2024; Peng et al. 2024; Mao et al. 2024; Duan et al. 2020; Y. Chen et al. 2021; Kim et al. 2014; Cha 2017; Im et al. 2022), while 8 studies did not mention related information (Kong et al. 2024; Deng et al. 2024; Hu et al. 2017; Mao et al. 2021; J. Chen et al. 2023; J. J. Zhang and Shi 2019; Ding et al. 2022; Shan et al. 2022). Among them, 9 studies reported no adverse events (Xie et al. 2021; Koch et al. 2019; S. R. Wang and Li 2022; Zhu et al. 2024; Peng et al. 2024; Mao et al. 2024; Y. Chen et al. 2021; Kim et al. 2014; Cha 2017). The remaining three studies reported adverse events such as chewing and neck muscle spasms caused by stimulation, nausea, mild or short‐lived headaches, dizziness, etc. However, no serious adverse events, such as seizures, occurred. Moreover, each of these adverse events occurred only once in their respective experimental groups (Liao et al. 2021; Duan et al. 2020; Im et al. 2022). It is noteworthy that among all the included studies, only one study had a dropout rate exceeding 15% (Cha 2017). Furthermore, no adverse events were observed during the follow‐up period. These results strongly demonstrate the good tolerability of cTMS.

## 4 Discussion

Balance and walking dysfunction are common physical motor dysfunctions after stroke, affecting approximately two‐thirds of patients with stroke (Veldema and Gharabaghi 2022). This study shows that cTMS can effectively improve balance and walking function in patients with stroke, enhancing the excitability of the corresponding cortical areas of the brain.

Analysis revealed that the BBS scores in the experimental group were significantly superior to those in the control group, and sensitivity analysis confirmed its effectiveness, providing strong evidence for the efficacy of cTMS in improving balance function among patients with stroke. The mechanism of balance control is complex and is regulated by a hierarchy of neural systems including the spinal cord, brainstem, cerebellum, basal ganglia and cerebral cortex (Fujimoto et al. 2014). As the center for motor control and learning, the cerebellum not only connects to higher centers (cerebral cortex, basal ganglia) to optimize motor precision and adaptability (Maas et al. 2020), but also directly regulates cortical descending fibers (Bostan et al. 2013; Matsugi and Okada 2020), which is crucial for posture, muscle tone, and motor coordination. In this study, the selected and stimulated cerebellar regions were mainly concentrated in the cerebellar hemispheres and the vermis of the cerebellum. These two parts have distinctive mechanisms of action in promoting the recovery of balance function in stroke patients. Specifically, most of the lateral cerebellar hemispheres belong to the “cerebrocerebellum” and are closely connected to the contralateral cerebral cortex (Coffman et al. 2011). They are responsible for processing information transmitted from the spinal cord and upper‐level brain structures, regulating the planning and initiation of body movements, and coordinating voluntary movements (Z. Liu 2020). Apart from the cerebellar hemispheres, the vermis of the cerebellum is a core region in trunk posture control. Most of the vermis of the cerebellum belongs to the “spinocerebellum” and receives somatosensory inputs from the spinal cord, especially signals from the trunk and the proximal parts of the limbs (Roostaei et al. 2014). It may serve as a candidate stimulation target for regulating the movement network related to balance.

Unlike previous studies, in performing subgroup analyses, we used the Bonferroni correction method. This correction method aims to avoid the occurrence of type I errors and control the probability of false‐positive results in the overall study to ensure the reliability and accuracy of the analyzed results. Refinement of the subgroup analyses revealed that the use of excitatory stimulation modalities (HF‐rTMS, iTBS) of moderate intensity (≤ 80% RMT) applied to the cerebellum during the subacute phase of stroke significantly improved BBS scores. This is attributed to the peak of neuroplasticity within 3 to 6 months after stroke, during which enhanced corticospinal excitability is closely related to improved motor function (Coleman et al. 2017; J. C. Chen and Shaw 2014). Therefore, enhanced motor cortical excitability is considered a marker of increased neuroplasticity, providing the necessary physiological basis for the reorganization of neuronal networks (Du et al. 2016). After 6 months of stroke onset, neuroplasticity enters a plateau phase for most patients, resulting in a tendency for the intervention effect of cTMS to weaken (Biernaskie et al. 2004, W. Zhang et al. 2023). Notably, our study delves deeper into the realm of stimulation intensity, differentiating from the research conducted by Wu et al. (2022), Zeng et al. (2024), J. Wang et al. (2024). The intensity of stimulation plays a pivotal role as it determines the depth of penetration, potentially impacting the efficacy of cTMS. Specifically, Purkinje cells receive inhibitory inputs from stellate and basket cells in the cerebellar molecular layer. Research by Harrington et al. suggests that low‐intensity cerebellar stimulation may affect the molecular layer in the superficial cerebellum, reducing intracortical inhibition and indirectly achieving an excitatory effect by inhibiting the inhibitory output of Purkinje cells to the cerebral cortex (Harrington and Hammond‐Tooke 2015). Furthermore, our results indicate that in both age groups (≤ 60 years and > 60 years), the BBS scores of the experimental group were significantly higher than those of the control group. This may suggest that cTMS has a certain therapeutic effect across different ages of patients.

For the lower limb motor function, data analysis showed that the 10MWT performance of the treatment group was significantly better than that of the control group, confirming the positive promoting effect of cTMS on the walking ability of patients with stroke. The improvement of walking speed, as the key to the recovery of walking function, is directly related to the quality of life and functional recovery of patients (Dickstein 2008). In addition, the reduction of step width reflects the enhancement of gait stability, which is an important basis for the improvement of walking speed, and the stimulation of the cerebellum may enhance the patients’ motor learning ability, which further improves the walking ability (Koch et al. 2019; Maas et al. 2020). Moreover, the improvement in FMA‐LE scores in the treatment group was superior to that in the control group, a finding consistent with previous Meta‐analysis results (Zeng et al. 2024). The stimulation site of cTMS mainly focuses on the lateral cerebellar hemisphere, which belongs to the “cerebro‐cerebellum” and has close structural connections with the contralateral cerebral cortex (Coffman et al. 2011). Based on this unique anatomical structure, the lateral cerebellar hemisphere and the contralateral cerebral cortex synergistically regulate voluntary movements of the limbs. Therefore, when the lateral cerebellar hemisphere is damaged, patients often exhibit limb coordination dysfunction (Coffman et al. 2011). The application of cTMS, by stimulating the lateral cerebellar hemisphere, may help restore this synergistic regulation mechanism, thereby effectively improving the limb motor ability of patients with stroke. For upper limb function, although the sample size was limited, descriptive analysis showed that cTMS significantly improved the upper limb sub‐scores (Mao et al. 2021). Stimulation of the dentate nucleus through the deep cerebellum was found to significantly enhance upper limb motor function in patients with chronic stroke, a process that is accompanied by increased neural reorganization and metabolism in the cerebral cortex (e.g., primary motor cortex, primary sensory cortex, and anterior auxiliary motor area, etc.), which are critical for fine control of the distal limb (Baker et al. 2023). Given the extensive projections of cerebellar representations of upper limb function, the future application of cTMS in the treatment of upper limb motor dysfunction in patients with stroke is promising.

This analysis demonstrates that cTMS can significantly enhance the MEP amplitude in patients with stroke. As an important indicator of cortical spinal tract excitability, MEP particularly reflects the functional state of the primary motor cortex (Rosso et al. 2022). The potential mechanism by which stimulating the cerebellum regulates related functional areas of the cerebral cortex is related to the “cerebellum‐thalamus‐cortex” circuit (Koch et al. 2019). Most of the information transfer between the cerebral cortex and the cerebellum is mediated by the dense connections between the efferent dentate nucleus‐thalamus‐cortex tract and the afferent cortex‐pontine‐cerebellar tract (Ramnani 2006; Palesi et al. 2017). Studies have shown that the dentate nucleus‐thalamus‐cortex pathway is a key route for the transmission of excitatory information from the cerebellum to the cerebral cortex, such as the primary motor cortex and the prefrontal cortex (França et al. 2018; Schulz et al. 2015). In the field of stroke rehabilitation, the application of cTMS is mainly based on the “interhemispheric competition model” (Ntakou et al. 2022). Since Purkinje cells in the cerebellum inhibit the dentate nucleus by releasing gamma‐aminobutyric acid, this weakens the excitatory output of the dentate nucleus to the cerebral cortex to a certain extent, an effect known as “cerebellar‐brain‐inhibition” (CBI) (Fernandez et al. 2018). By utilizing the CBI effect and adjusting the stimulation parameters of TMS, it is possible to assess the integrity of the cerebellum‐brain pathway and regulate the excitability level of the contralateral cerebral hemisphere (Fernandez et al. 2018; Ugawa et al. 1995). For example, iTBS can increase the MEP amplitude in the contralateral cerebral hemisphere, while cTBS produces the opposite effect (Koch et al. 2008), thereby enabling the regulation of cortical excitability in the cerebral hemispheres.

Results of follow‐up show that, compared with the control group, the effects of cTMS in improving the balance and limb motor function of the experimental group are inconsistent. In the TUG test, we did not observe significant improvement, which is likely attributed to the fact that the TUG test assesses not only balance ability but also functional mobility (Hafsteinsdóttir et al. 2014). Similarly, there was no significant increase in the FMA–LE score, which may imply that during the follow‐up period, the recovery of patients’ limb motor function relied more on conventional rehabilitation treatments (J. Chen et al. 2023). Clinically, the importance of the sustained efficacy of non‐invasive brain‐stimulation techniques is self‐evident, and it is one of the key factors for their widespread application (Pisegna et al. 2016). However, based on the current research, we have not observed the exact long‐term efficacy of cTMS. This may be due to the limited number of studies or the relatively short treatment cycles. Therefore, it is necessary to conduct further research in the future to longitudinally observe the long‐term efficacy of cTMS.

### 4.1 Strength

1. This meta‐analysis incorporated the most recent researches available. In contrast to past meta‐analyses, such as those conducted by Wu et al. (2022), which synthesized relevant articles up to October 2021, and by Zeng et al. (2024) and J. Wang et al. (2024), which summarized the relevant research up to 2023, our study has included numerous new studies published over the past year (Zhu et al. 2024; Kong et al. 2024; Peng et al. 2024; Mao et al. 2024; Deng et al. 2024). This comprehensive inclusion of the latest research ensures that our findings are based on the most up‐to‐date evidence.
2. This study further explored the specific effects of different stroke phases, treatment modes, intensities, and total interventional sessions on the therapeutic efficacy of cTMS for balance. Notably, our study delves deeper into the realm of stimulation intensity, differentiating from the previous meta‐analyses (Wu et al. 2022; Zeng et al. 2024; J. Wang et al. 2024). The intensity of stimulation plays a pivotal role as it determines the depth of penetration, potentially impacting the efficacy of cTMS.
3. To ensure the reliability and accuracy of our subgroup analysis results, we employed the Bonferroni correction method. This rigorous approach is unique compared to previous meta‐analyses and helps avoid Type I errors. Refinement of the subgroup analyses revealed that the use of excitatory stimulation modalities (HF‐rTMS, iTBS) of moderate intensity (≤ 80% RMT) applied to the cerebellum during the subacute phase of stroke significantly improved BBS scores. Furthermore, we observed that 5 to 10 sessions of cTMS notably improved patients’ BBS scores, with the effect size increasing as the number of treatment sessions rose. Similarly, J. Wang et al. (2024) found that the effect size of more than 10 treatment sessions was greater than that of 10 or fewer sessions. This discovery may be linked to the long‐term potentiation induced by TMS. Due to its positive feedback nature, synaptic plasticity can be an unstable process–the more enhancement induced, the more related activity generated, which in turn leads to further enhancement (Pell et al. 2011).

### 4.2 Limitations

This study has certain limitations: First, while the impact of key factors such as medication use, lesion location, and severity on the functional outcomes of patients with stroke is widely recognized, not all included studies have documented these factors in detail, which affects the applicability of cTMS. Second, variations in cTMS treatment parameters among studies have led to significant heterogeneity in some outcomes. Although sensitivity analysis, subgroup analysis, and Bonferroni correction have been employed to ensure the robustness of the results, these variations still pose challenges for comparing outcomes and formulating standardized treatment guidelines. Therefore, there is an urgent need for future research to conduct large‐sample, high‐quality RCTs to directly compare the specific effects of different treatment parameters on cTMS for patients with stroke. Furthermore, inconsistencies in randomization and allocation concealment methods may introduce randomization bias, and the lack of assessor blinding in some studies increases the risk of outcome measurement bias, thereby affecting the objectivity of the results. To address this, we further examined the credibility of the outcomes using the GRADE system. Additionally, the lack of follow‐up in most studies undermines the reliability of the long‐term effects of cTMS. Meanwhile, not all studies have comprehensively reported information related to adverse events, including details such as the severity and duration of adverse reactions, which to some extent limits our ability to conduct a thorough assessment of treatment safety. Finally, the fact that the included literature is mainly sourced from Chinese or English may lead to language‐based selection bias, affecting the comprehensiveness and representativeness of the results.

## 5 Conclusion

Combining the results of meta‐analysis and subgroup analysis, cTMS can effectively improve the motor function of patients with stroke, with significant efficacy demonstrated in balance and limb motor function. Subgroup analyses showed that the therapeutic effect of cTMS was more pronounced when intervening in the subacute phase of stroke, and when the excitatory stimulation mode and moderate intensity (≤ 80% RMT) were used. Further clinical studies could explore more specific stimulation parameters and treatment protocols to further optimize the application of cTMS. In summary, cTMS emerges as a rapid and effective rehabilitation therapy adjunct, offering new hope and prospects for the rehabilitation of motor dysfunction in patients with stroke.

## Author Contributions

**Yongxin Zhu**: conceptualization, data curation, formal analysis, investigation, methodology, project administration, resources, software, validation, visualization, writing – original draft, writing – review and editing. **Juncong Yang**: data curation, formal analysis, investigation, methodology, project administration, resources, software, validation. **Kun Wang**: methodology, validation, writing – review and editing. **Xianwen Li**: conceptualization, methodology. **Jiahui Ling**: data curation, methodology, resources. **Xie Wu**: methodology, supervision. **Lianhui Fu**: project administration, supervision. **Qi Qi**: conceptualization, funding acquisition, project administration, supervision, writing – review and editing.

## Conflicts of Interest

The authors declare no conflicts of interest.

### Peer Review

The peer review history for this article is available at https://publons.com/publon/10.1002/brb3.70471

## Data Availability

The data that support the findings of this study are available from the corresponding author upon reasonable request.

## Search strategy

**Table:**

Database		Search terms	Records
Web of Science	**1**	**ALL = Stroke**	1464
	**2**	**ALL = (Cerebrovascular Accident) OR (CVA) OR (Cerebrovascular Apoplexy) OR (Brain Vascular Accident) OR (Cerebrovascular Stroke) OR (Apoplexy) OR (Cerebral Stroke) OR (Acute Stroke) OR (Acute Cerebrovascular Accident) OR (Brain Infarction) OR (Anterior Circulation Brain Infarction) OR (Brain Venous Infarction) OR (Posterior Circulation Brain Infarction) OR (Hemorrhagic Stroke) OR (Subarachnoid Hemorrhagic Stroke) OR (Intracerebral Hemorrhagic Stroke) OR (Ischemic Stroke) OR (Cryptogenic Ischemic Stroke) OR (Wake‐up Stroke) OR (Acute Ischemic Stroke)**	
	**3**	**#1 OR #2**	
	**4**	**ALL = Transcranial Magnetic Stimulation**	
	**5**	**ALL = (transcranial magnetic stimulat*) OR (TMS) OR (repetitive transcranial magnetic stimulat*) OR (repetitive transcranial magnetic stimulation) OR (rTMS) OR (theta burst stimulation) OR (theta burst stimulat*) OR (TBS) OR (stimulation) OR (stimulat*)**	
	**6**	**#4 OR #5**	
	**7**	**ALL = Cerebellum**	
	**8**	**ALL = (Corpus Cerebelli) OR (Cerebella) OR (proencephalon) OR (cerebellar)**	
	**9**	**#7 OR #8**	
	**10**	**#3 AND #6 AND #9**	
PubMed	**1**	**“Stroke”[Mesh]**	493
	**2**	**(Cerebrovascular Accident) OR (CVA) OR (Cerebrovascular Apoplexy) OR (Brain Vascular Accident) OR (Cerebrovascular Stroke) OR (Apoplexy) OR (Cerebral Stroke) OR (Acute Stroke) OR (Acute Cerebrovascular Accident) OR (“Brain Infarction”[Mesh]) OR (Anterior Circulation Brain Infarction) OR (Brain Venous Infarction) OR (Posterior Circulation Brain Infarction) OR (“Hemorrhagic Stroke”[Mesh]) OR (Subarachnoid Hemorrhagic Stroke) OR (Intracerebral Hemorrhagic Stroke) OR (“Ischemic Stroke”[Mesh]) OR (Cryptogenic Ischemic Stroke) OR (Wake‐up Stroke) OR (Acute Ischemic Stroke) **	
	**3**	**#1 OR #2**	
	**4**	**“Transcranial Magnetic Stimulation”[Mesh]**	
	**5**	**(transcranial magnetic stimulat*) OR (TMS) OR (repetitive transcranial magnetic stimulat*) OR (repetitive transcranial magnetic stimulation) OR (rTMS) OR (theta burst stimulation) OR (theta burst stimulat*) OR (TBS) OR (stimulation) OR (stimulat*)**	
	**6**	**#4 OR #5**	
	**7**	**“Cerebellum”[Mesh]**	
	**8**	**(Corpus Cerebelli) OR (Cerebella) OR (Parencephalon) OR (cerebellar)**	
	**9**	**#7 OR #8**	
	**10**	**#3 AND #6 AND #9**	
The Cochrane Library	**#1**	**Stroke:ti, ab, kw**	190
	**#2**	**(Cerebrovascular Accident) OR (CVA) OR (Cerebrovascular Apoplexy) OR (Brain Vascular Accident) OR (Cerebrovascular Stroke) OR (Apoplexy) OR (Cerebral Stroke) OR (Acute Stroke) OR (Acute Cerebrovascular Accident) OR (Brain Infarction) OR (Anterior Circulation Brain Infarction) OR (Brain Venous Infarction) OR (Posterior Circulation Brain Infarction) OR (Hemorrhagic Stroke) OR (Subarachnoid Hemorrhagic Stroke) OR (Intracerebral Hemorrhagic Stroke) OR (Ischemic Stroke) OR (Cryptogenic Ischemic Stroke) OR (Wake‐up Stroke) OR (Acute Ischemic Stroke):ti, ab, kw**	
	**#3**	**#1 OR #2**	
	**#4**	**Transcranial Magnetic Stimulation:ti, ab, kw**	
	**#5**	**(transcranial magnetic stimulat*) OR (TMS) OR (repetitive transcranial magnetic stimulat*) OR (repetitive transcranial magnetic stimulation) OR (rTMS) OR (theta burst stimulation) OR (theta burst stimulat*) OR (TBS) OR (stimulation) OR (stimulat*):ti, ab, kw**	
	**#6**	**#4 OR #5**	
	**#7**	**Cerebellum:ti, ab, kw**	
	**#8**	**(Corpus Cerebelli) OR (Cerebella) OR (proencephalon) OR (cerebellar):ti, ab, kw**	
	**#9**	**#7 OR #8**	
	**#10**	**#3 AND #6 AND #9**	
Embase	**#1**	**‘cerebrovascular accident’/exp**	908
	**#2**	**‘accident, cerebrovascular’ OR ‘acute cerebrovascular lesion’ OR ‘acute focal cerebral vasculopathy’ OR ‘acute stroke’ OR ‘apoplectic stroke’ OR ‘apoplexia’ OR ‘apoplexy’ OR ‘blood flow disturbance, brain’ OR ‘brain accident’ OR ‘brain attack’ OR ‘brain blood flow disturbance’ OR ‘brain insult’ OR ‘brain insultus’ OR ‘brain vascular accident’ OR ‘cerebral apoplexia’ OR ‘cerebral insult’ OR ‘cerebral stroke’ OR ‘cerebral vascular accident’ OR ‘cerebral vascular insufficiency’ OR ‘cerebro vascular accident’ OR ‘cerebrovascular arrest’ OR ‘cerebrovascular failure’ OR ‘cerebrovascular injury’ OR ‘cerebrovascular insufficiency’ OR ‘cerebrovascular insult’ OR ‘cerebrum vascular accident’ OR ‘cryptogenic stroke’ OR ‘cva’ OR ‘insultus cerebralis’ OR ‘ischaemic seizure’ OR ‘ischemic seizure’ OR ‘stroke’ OR ‘thrombotic stroke’ OR ‘cerebrovascular accident’**	
	**#3**	**#1 OR #2**	
	**#4**	**‘transcranial magnetic stimulation’/exp**	
	**#5**	**‘magnetic stimulation, transcranial’ OR ‘stimulation, transcranial magnetic’ OR ‘transcranial magnetic stimulation’ OR ‘repetitive transcranial magnetic stimulation’/exp OR ‘transcranial magnetic stimulation, repetitive’ OR ‘repetitive transcranial magnetic stimulation’ OR tms OR (transcranial AND magnetic AND stimulat*) OR (repetitive AND transcranial AND magnetic AND stimulat*) OR rtms OR (theta AND burst AND stimulation) OR (theta AND burst AND stimulat*) OR tbs OR stimulation OR stimulat***	
	**#6**	**#4 OR #5**	
	**#7**	**‘cerebellum’/exp**	
	**#8**	**‘cerebellar organisation’ OR ‘cerebellar organization’ OR ‘cerebellum’ OR (corpus AND cerebelli) OR cerebella OR parencephalon OR cerebellar**	
	**#9**	**#7 OR #8**	
	**#10**	**#3 AND #6 AND #9**	
CNKI	**#1**	**SU = 卒中 + 中风 + 脑血管意外 + 脑梗 + 缺血性脑梗死 + 脑出血 + 出血性脑梗死**	380
	**#2**	**SU = 经颅磁刺激 + 重复经颅磁刺激 + 间歇性θ短阵脉冲刺激 + 持续性θ短阵脉冲刺激 + θ短阵快速脉冲经颅磁刺激 + θ脉冲刺激 + 刺激 + TMS + TBS**	
	**#3**	**SU = 小脑**	
	**#4**	**#1 AND #2 AND #3**	
Wanfang	**#1**	**主题 = 卒中 or 中风 or 脑血管意外 or 脑梗 or 缺血性脑梗死 or 脑出血 or 出血性脑梗死**	527
	**#2**	**主题 = 经颅磁刺激 or 重复经颅磁刺激 or 间歇性θ短阵脉冲刺激 or 持续性θ短阵脉冲刺激 or θ短阵快速脉冲经颅磁刺激 or θ脉冲刺激 or 刺激 or TMS or TBS**	
	**#3**	**主题 = 小脑**	
	**#4**	**#1 AND #2 AND #3**	

## References

[brb370471-bib-0001] Baker, K. B. , E. B. Plow , S. Nagel , et al. 2023. “Cerebellar Deep Brain Stimulation for Chronic Post‐Stroke Motor Rehabilitation: A Phase I Trial.” Nature Medicine 29, no. 9: 2366–2374. 10.1038/s41591-023-02507-0 PMC10504081 37580534

[brb370471-bib-0002] Balshem, H. , M. Helfand , H. J. Schünemann , et al. 2011. “GRADE Guidelines: 3. Rating the Quality of Evidence.” Journal of Clinical Epidemiology 64, no. 4: 401–406. 21208779 10.1016/j.jclinepi.2010.07.015

[brb370471-bib-0003] Biernaskie, J. , G. Chernenko , and D Corbett . 2004. “Efficacy of Rehabilitative Experience Declines With Time After Focal Ischemic Brain Injury.” Journal of Neuroscience 24, no. 5: 1245–1254. 14762143 10.1523/JNEUROSCI.3834-03.2004 PMC6793570

[brb370471-bib-0004] Bostan, A. C. , R. P. Dum , and P. L Strick . 2010. “The Basal Ganglia Communicate With the Cerebellum.” Proceedings of the National Academy of Sciences of the United States of America 107, no. 18: 8452–8456. 20404184 10.1073/pnas.1000496107 PMC2889518

[brb370471-bib-0005] Bostan, A. C. , R. P. Dum , and P. L Strick . 2013. “Cerebellar Networks With the Cerebral Cortex and Basal Ganglia.” Trends in Cognitive Sciences 17, no. 5: 241–254. 23579055 10.1016/j.tics.2013.03.003 PMC3645327

[brb370471-bib-0006] Cha, H. G. 2017. “The Effect of Low‐Frequency (1 Hz) rTMS on the Cerebellar Cortex in Patients With Ataxia After a Posterior Circulation Stroke: Randomized Control Trial.” Journal of Magnetics 22, no. 4: 625–629.

[brb370471-bib-0007] Chen, J. , M. F. Shi , J. Chen , et al. 2023. “The Effects of Supplementing Theta Burst Stimulation of the Cerebellum With Physical Therapy on Balance and Gait Recovery After a Stroke: A Randomized Clinical Trial.” Chinese Journal of Physical Medicine and Rehabilitation 45, no. 5: 402–407.

[brb370471-bib-0008] Chen, J. C. , and F. Z Shaw . 2014. “Progress in Sensorimotor Rehabilitative Physical Therapy Programs for Stroke Patients.” World Journal of Clinical Cases 2, no. 8: 316–326. 25133141 10.12998/wjcc.v2.i8.316 PMC4133420

[brb370471-bib-0009] Chen, Y. , Q. C. Wei , M. Z. Zhang , et al. 2021. “Cerebellar Intermittent Theta‐Burst Stimulation Reduces Upper Limb Spasticity after Subacute Stroke: A Randomized Controlled Trial.” Front Neural Circuits 15: 655502. 34776874 10.3389/fncir.2021.655502 PMC8578104

[brb370471-bib-0010] Coffman, K. A. , R. P. Dum , and P. L Strick . 2011. “Cerebellar Vermis Is a Target of Projections From the Motor Areas in the Cerebral Cortex.” Proceedings of the National Academy of Sciences of the United States of America 108, no. 38: 16068–16073. 21911381 10.1073/pnas.1107904108 PMC3179064

[brb370471-bib-0011] Coleman, E. R. , R. Moudgal , K. Lang , et al. 2017. “Early Rehabilitation After Stroke: A Narrative Review.” Current Atherosclerosis Reports 19, no. 12: 59. 29116473 10.1007/s11883-017-0686-6 PMC5802378

[brb370471-bib-0012] Deng, L. Y. , Y. Chen , N. Zeng , et al. 2024. “Effect of Contralesional Cerebellar iTBS Combined With Routine Rehabilitation on Lower Limb Walking Function in Stroke Patients.” Journal of Practical Medicine 40, no. 13: 1797–1802.

[brb370471-bib-0013] Dickstein, R. 2008. “Rehabilitation of Gait Speed After Stroke: A Critical Review of Intervention Approaches.” Neurorehabilitation and Neural Repair 22, no. 6: 649–660. 18971380 10.1177/1545968308315997

[brb370471-bib-0014] Ding, X. C. , J. Yuan , J. Chen , et al. 2022. “Effects of Repetitive Transcranial Magnetic Stimulation of the Cerebellum on Walking Disorder, Balance Function, and Magnetic Resonance Spectroscopy Indexes in Patients With Ischemic Stroke.” Hainan Medical Journal 33, no. 6: 688–691.

[brb370471-bib-0015] Du, J. , L. Tian , W. Liu , et al. 2016. “Effects of Repetitive Transcranial Magnetic Stimulation on Motor Recovery and Motor Cortex Excitability in Patients With Stroke: A Randomized Controlled Trial.” European Journal of Neurology 23, no. 11: 1666–1672. 27425785 10.1111/ene.13105

[brb370471-bib-0016] Duan, Q. , L. W. Sun , C. X. Wei , et al. 2020. “Effect of Cerebellar Low Frequency rTMS on Lower Lamb Motor Function and Cortical Excitability in Patients With Posterior Circulation Stroke.” Chinese Journal of Brain Diseases and Rehabilitation 10, no. 6: 352–356.

[brb370471-bib-0017] Duncan, P. W. , R. Zorowitz , B. Bates , et al. 2005. “Management of Adult Stroke Rehabilitation Care: A Clinical Practice Guideline.” Stroke; A Journal of Cerebral Circulation 36, no. 9: e100–e143. 10.1161/01.STR.0000180861.54180.FF 16120836

[brb370471-bib-0018] Fernandez, L. , B. P. Major , W. P. Teo , L. K. Byrne , and P. G Enticott . 2018. “Assessing Cerebellar Brain Inhibition (CBI) via Transcranial Magnetic Stimulation (TMS): a Systematic Review.” Neuroscience and Biobehavioral Reviews 86: 176–206. 29208533 10.1016/j.neubiorev.2017.11.018

[brb370471-bib-0019] Fisicaro, F. , G. Lanza , A. A. Grasso , et al. 2019. “Repetitive Transcranial Magnetic Stimulation in Stroke Rehabilitation: Review of the Current Evidence and Pitfalls.” Therapeutic Advances in Neurological Disorders 12: 1756286419878317. 31598137 10.1177/1756286419878317 PMC6763938

[brb370471-bib-0020] França, C. , D. C. de Andrade , M. J. Teixeira , et al. 2018. “Effects of Cerebellar Neuromodulation in Movement Disorders: A Systematic Review.” Brain Stimulation 11, no. 2: 249–260. 29191439 10.1016/j.brs.2017.11.015

[brb370471-bib-0021] Fujimoto, H. , M. Mihara , N. Hattori , et al. 2014. “Cortical Changes Underlying Balance Recovery in Patients With Hemiplegic Stroke.” Neuroimage 85, no. Pt 1: 547–554. 23684871 10.1016/j.neuroimage.2013.05.014

[brb370471-bib-0022] GBD 2019 Stroke Collaborators . 2021. “Global, Regional, and National Burden of Stroke and Its Risk Factors, 1990–2019: A Systematic Analysis for the Global Burden of Disease Study 2019.” Lancet Neurology 20, no. 10: 795–820. 34487721 10.1016/S1474-4422(21)00252-0 PMC8443449

[brb370471-bib-0023] Gorst, T. , A. Rogers , S. C. Morrison , et al. 2019. “The Prevalence, Distribution, and Functional Importance of Lower Limb Somatosensory Impairments in Chronic Stroke Survivors: A Cross Sectional Observational Study.” Disability and Rehabilitation 41, no. 20: 2443–2450. 29726732 10.1080/09638288.2018.1468932

[brb370471-bib-0024] Hafsteinsdóttir, T. B. , M. Rensink , and M Schuurmans . 2014. “Clinimetric Properties of the Timed up and Go Test for Patients With Stroke: A Systematic Review.” Topics in Stroke Rehabilitation 21, no. 3: 197–210. 24985387 10.1310/tsr2103-197

[brb370471-bib-0025] Hallett, M. 2007. “Transcranial Magnetic Stimulation: A Primer.” Neuron 55, no. 2: 187–199. 17640522 10.1016/j.neuron.2007.06.026

[brb370471-bib-0026] Harrington, A. , and G. D Hammond‐Tooke . 2015. “Theta Burst Stimulation of the Cerebellum Modifies the TMS‐Evoked N100 Potential, a Marker of GABA Inhibition.” PLoS ONE 10, no. 11: e0141284. 26529225 10.1371/journal.pone.0141284 PMC4631469

[brb370471-bib-0027] Hu, X. H. , W. Y. Yang , R. Guo , et al. 2017. “Clinical Observation on Low Frequency Repetitive Transcranial Magnetic Stimulation for the Treatment of Balance Disorders After Cerebellar Infarction.” Chinese Journal of Integrative Medicine on Cardio‐/Cerebrovascular Disease 15, no. 17: 2107–2109.

[brb370471-bib-0028] Im, N. G. , K. R. Oh , M. G. Kim , et al. 2022. “Effect of Low Frequency Cerebellar Repetitive Transcranial Magnetic Stimulation on Balance Impairment in Patients with Cerebral Infarction.” Annals of Rehabilitation Medicine 46, no. 6: 275–283. 36588442 10.5535/arm.22058 PMC9810654

[brb370471-bib-0029] Kang, N. , R. D. Lee , J. H. Lee , and M. H Hwang . 2020. “Functional Balance and Postural Control Improvements in Patients With Stroke after Noninvasive Brain Stimulation: A Meta‐Analysis.” Archives of Physical Medicine and Rehabilitation 101, no. 1: 141–153. 31568760 10.1016/j.apmr.2019.09.003

[brb370471-bib-0030] Kim, W. S. , S. H. Jung , M. K. Oh , Y. S. Min , J. Y. Lim , and N. J Paik . 2014. “Effect of Repetitive Transcranial Magnetic Stimulation Over the Cerebellum on Patients With Ataxia After Posterior Circulation Stroke: a Pilot Study.” Journal of Rehabilitation Medicine 46, no. 5: 418–423. 24658396 10.2340/16501977-1802

[brb370471-bib-0031] Koch, G. , S. Bonnì , E. P. Casula , et al. 2019. “Effect of Cerebellar Stimulation on Gait and Balance Recovery in Patients with Hemiparetic Stroke: a Randomized Clinical Trial.” JAMA Neurology 76, no. 2: 170–178. 30476999 10.1001/jamaneurol.2018.3639 PMC6439971

[brb370471-bib-0032] Koch, G. , F. Mori , B. Marconi , et al. 2008. “Changes in Intracortical Circuits of the human Motor Cortex Following Theta Burst Stimulation of the Lateral Cerebellum.” Clinical Neurophysiology 119, no. 11: 2559–2569. 18824403 10.1016/j.clinph.2008.08.008

[brb370471-bib-0033] Kong, Q. , Z. L. Guo , C. F. Gao , et al. 2024. “Intermittent Theta Burst Stimulation of the Cerebellum Can Improve the Walking of Stroke Survivors With Lower Limb Dysfunction.” Chinese Journal of Physical Medicine and Rehabilitation 46, no. 3: 226–231.

[brb370471-bib-0034] Lefaucheur, J. P. , A. Aleman , C. Baeken , et al. 2020. “Evidence‐Based Guidelines on the Therapeutic Use of Repetitive Transcranial Magnetic Stimulation (rTMS): An Update (2014‐2018).” Clinical Neurophysiology 131, no. 2: 474–528. 31901449 10.1016/j.clinph.2019.11.002

[brb370471-bib-0035] Li, J. , P. Zhang , W. Tao , X. Yi , J. Zhang , and C Wang . 2018. “Age‐Specific Clinical Characteristics and Outcome in Patients Over 60 Years Old With Large Hemispheric Infarction.” Brain and Behavior 8, no. 12: e01158. 30566281 10.1002/brb3.1158 PMC6305916

[brb370471-bib-0036] Liao, L. Y. , Y. J. Xie , Y. Chen , and Q Gao . 2021. “Cerebellar Theta‐Burst Stimulation Combined with Physiotherapy in Subacute and Chronic Stroke Patients: A Pilot Randomized Controlled Trial.” Neurorehabilitation and Neural Repair 35, no. 1: 23–32. 33166213 10.1177/1545968320971735

[brb370471-bib-0037] Liao, L. Y. , Y. Zhu , Q. Y. Peng , et al. 2024. “Intermittent Theta‐Burst Stimulation for Stroke: Primary Motor Cortex versus Cerebellar Stimulation: A Randomized Sham‐Controlled Trial.” Stroke; A Journal of Cerebral Circulation 55, no. 1: 156–165. 10.1161/STROKEAHA.123.044892 38037225

[brb370471-bib-0038] Liu, Q. , Y. Liu , and Y Zhang . 2024. “Effects of Cerebellar Non‐Invasive Stimulation on Neurorehabilitation in Stroke Patients: An Updated Systematic Review.” Biomedicines 12, no. 6:. 10.3390/biomedicines12061348 PMC11201496 38927555

[brb370471-bib-0039] Liu, Z. 2020. “Implications of New Findings on Functions of the Cerebellum for Motor Skill Learning and Performance From Neuroscience Perspectives.” China Sport Science and Technology 56, no. 01: 45–54.

[brb370471-bib-0040] Maas, R. , R. C. G. Helmich , and B. P. C van de Warrenburg . 2020. “The Role of the Cerebellum in Degenerative Ataxias and Essential Tremor: Insights From Noninvasive Modulation of Cerebellar Activity.” Movement Disorders 35, no. 2: 215–227. 31820832 10.1002/mds.27919 PMC7027854

[brb370471-bib-0041] Mao, J. N. , L. H. Cui , L. Chang , et al. 2021. “Analysis of the Effect of Repetitive Transcranial Magnetic Stimulation of the Cerebellum on the Balance Function of Stroke Patients.” RARM 2, no. 4: 146–149.

[brb370471-bib-0042] Mao, J. N. , H. Zhang , and L. H Cui . 2024. “The Effect of Cerebellar Transcranial Magnetic Stimulation on Balance and Motor Function in Patients With Hemiplegia Following Stroke.” Neural Injury and Functional Reconstruction 19, no. 10: 574–578+604.

[brb370471-bib-0043] Matsugi, A. , and Y Okada . 2020. “Cerebellar Transcranial Direct Current Stimulation Modulates the Effect of Cerebellar Transcranial Magnetic Stimulation on the Excitability of Spinal Reflex.” Neuroscience Research 150: 37–43. 30794822 10.1016/j.neures.2019.01.012

[brb370471-bib-0044] Meseguer‐Henarejos, A. B. , M. Rubio‐Aparicio , J. A. López‐Pina , R. Carles‐Hernández , and A Gómez‐Conesa . 2019. “Characteristics That Affect Score Reliability in the Berg Balance Scale: A Meta‐Analytic Reliability Generalization Study.” European Journal of Physical and Rehabilitation Medicine 55, no. 5: 570–584. 30955319 10.23736/S1973-9087.19.05363-2

[brb370471-bib-0045] Ntakou, E. A. , G. Nasios , A. Nousia , V. Siokas , L. Messinis , and E Dardiotis . 2022. “Targeting Cerebellum With Non‐Invasive Transcranial Magnetic or Current Stimulation After Cerebral Hemispheric Stroke‐Insights for Corticocerebellar Network Reorganization: a Comprehensive Review.” Healthcare 10, no. 12: 2401. 36553925 10.3390/healthcare10122401 PMC9778071

[brb370471-bib-0046] O'Reilly, J. X. , C. F. Beckmann , V. Tomassini , N. Ramnani , and H Johansen‐Berg . 2010. “Distinct and Overlapping Functional Zones in the Cerebellum Defined by Resting state Functional Connectivity.” Cerebral Cortex 20, no. 4: 953–965. 19684249 10.1093/cercor/bhp157 PMC2837094

[brb370471-bib-0047] Palesi, F. , A. De Rinaldis , G. Castellazzi , et al. 2017. “Contralateral Cortico‐ponto‐Cerebellar Pathways Reconstruction in Humans In Vivo: Implications for Reciprocal Cerebro‐Cerebellar Structural Connectivity in Motor and Non‐Motor Areas.” Scientific Reports 7, no. 1: 12841. 28993670 10.1038/s41598-017-13079-8 PMC5634467

[brb370471-bib-0048] Pell, G. S. , Y. Roth , and A Zangen . 2011. “Modulation of Cortical Excitability Induced by Repetitive Transcranial Magnetic Stimulation: Influence of Timing and Geometrical Parameters and Underlying Mechanisms.” Progress in Neurobiology 93, no. 1: 59–98. 21056619 10.1016/j.pneurobio.2010.10.003

[brb370471-bib-0049] Peng, J. , S. Xie , Y. Cha , et al. 2024. “Effects of Intermittent Theta Short Burst Pulse Stimulation to the Cerebellum Vermis on Lower Limb Motor Dysfunction in Stroke Patients.” Neural Injury and Functional Reconstruction 20, no. 1: 21–25.

[brb370471-bib-0050] Pisegna, J. M. , A. Kaneoka , W. G. Pearson , Jr., S. Kumar , and S. E Langmore . 2016. “Effects of Non‐Invasive Brain Stimulation on Post‐Stroke Dysphagia: a Systematic Review and Meta‐Analysis of Randomized Controlled Trials.” Clinical Neurophysiology 127, no. 1: 956–968. 26070517 10.1016/j.clinph.2015.04.069 PMC5326549

[brb370471-bib-0051] Ramnani, N. 2006. “The Primate Cortico‐Cerebellar System: Anatomy and Function.” Nature Reviews Neuroscience 7, no. 7: 511–522. 16791141 10.1038/nrn1953

[brb370471-bib-0052] Rastogi, A. , R. Cash , K. Dunlop , et al. 2017. “Modulation of Cognitive Cerebello‐Cerebral Functional Connectivity by Lateral Cerebellar Continuous Theta Burst Stimulation.” Neuroimage 158: 48–57. 28669908 10.1016/j.neuroimage.2017.06.048

[brb370471-bib-0053] Roostaei, T. , A. Nazeri , M. A. Sahraian , and A Minagar . 2014. “The human Cerebellum: A Review of Physiologic Neuroanatomy.” Neurologic Clinics 32, no. 4: 859–869. 25439284 10.1016/j.ncl.2014.07.013

[brb370471-bib-0054] Rosso, C. , E. J. Moulton , C. Kemlin , et al. 2022. “Cerebello‐Motor Paired Associative Stimulation and Motor Recovery in Stroke: A Randomized, Sham‐Controlled, Double‐Blind Pilot Trial.” Neurotherapeutics 19, no. 2: 491–500. 35226342 10.1007/s13311-022-01205-y PMC9226244

[brb370471-bib-0055] Schulz, R. , M. J. Wessel , M. Zimerman , J. E. Timmermann , C. Gerloff , and F. C Hummel . 2015. “White Matter Integrity of Specific Dentato‐Thalamo‐Cortical Pathways Is Associated With Learning Gains in Precise Movement Timing.” Cerebral Cortex 25, no. 7: 1707–1714. 24443417 10.1093/cercor/bht356

[brb370471-bib-0056] Shan, X. C. , P. Zhou , Y. Zhang , et al. 2022. “Clinical Observation on Cluster Needling of Scalp Points Combined With Repeated Transcranial Magnetic Stimulation in Treating Balance Disorder After Cerebellar Infarction.” China Medical Herald 19, no. 20: 119–122.

[brb370471-bib-0057] Singer, B. , and J Garcia‐Vega . 2017. “The Fugl‐Meyer Upper Extremity Scale.” Journal of Physiotherapy 63, no. 1: 53. 27964964 10.1016/j.jphys.2016.08.010

[brb370471-bib-0058] Tu, W. J. , Z. Zhao , P. Yin , et al. 2023. “Estimated Burden of Stroke in China in 2020.” JAMA Network Open 6, no. 3: e231455. 36862407 10.1001/jamanetworkopen.2023.1455 PMC9982699

[brb370471-bib-0059] Ugawa, Y. , Y. Uesaka , Y. Terao , R. Hanajima , and I Kanazawa . 1995. “Magnetic Stimulation Over the Cerebellum in Humans.” Annals of Neurology 37, no. 6: 703–713. 7778843 10.1002/ana.410370603

[brb370471-bib-0060] Van Criekinge, T. , C. Heremans , J. Burridge , et al. 2024. “Standardized Measurement of Balance and Mobility Post‐Stroke: Consensus‐Based Core Recommendations From the Third Stroke Recovery and Rehabilitation Roundtable.” Neurorehabilitation and Neural Repair 38, no. 1: 41–51. 37837351 10.1177/15459683231209154

[brb370471-bib-0061] Veldema, J. , and A Gharabaghi . 2022. “Non‐Invasive Brain Stimulation for Improving Gait, Balance, and Lower Limbs Motor Function in Stroke.” Journal of Neuroengineering and Rehabilitation 19, no. 1: 84. 35922846 10.1186/s12984-022-01062-y PMC9351139

[brb370471-bib-0062] Wang, J. , Z. Wu , S. Hong , et al. 2024. “Cerebellar Transcranial Magnetic Stimulation for Improving Balance Capacity and Activity of Daily Living in Stroke Patients: A Systematic Review and Meta‐Analysis.” BMC Neurology 24, no. 1: 205. 38879485 10.1186/s12883-024-03720-1 PMC11179288

[brb370471-bib-0063] Wang, S. R. , and L Li . 2022. “Effects of Cerebellar Theta‐Burst Stimulation on Lower Extremity Motor Function in Stroke Patients.” Chinese Journal of Rehabilitation Theory and Practice 28, no. 10: 1205–1210.

[brb370471-bib-0064] Ward, N. S. 2004. “Functional Reorganization of the Cerebral Motor System After Stroke.” Current Opinion in Neurology 17, no. 6: 725–730. 15542982 10.1097/00019052-200412000-00013

[brb370471-bib-0065] Wu, Z. Y. , Y. Q. Wang , X. P. Wen , et al. 2022. “Does Noninvasive Cerebellar Stimulation Improve the Balance and Walking Function of Patients With Stroke: A Meta‐Analysis of Randomized Controlled Trials.” Medicine 101, no. 36: e30302. 36086722 10.1097/MD.0000000000030302 PMC10980459

[brb370471-bib-0066] Xie, Y. J. , Q. C. Wei , Y. Chen , et al. 2021. “Cerebellar Theta Burst Stimulation on Walking Function in Stroke Patients: A Randomized Clinical Trial.” Frontiers in neuroscience 15: 688569. 34764848 10.3389/fnins.2021.688569 PMC8576464

[brb370471-bib-0067] Zeng, Y. , Z. Ye , W. Zheng , and J Wang . 2024. “Efficacy of Cerebellar Transcranial Magnetic Stimulation for Post‐Stroke Balance and Limb Motor Function Impairments: Meta‐Analyses of Random Controlled Trials and Resting‐State fMRI Studies.” Cerebellum 23, no. 4: 1678–1696. 38280142 10.1007/s12311-024-01660-7

[brb370471-bib-0068] Zhang, J. J. , and Y. H Shi . 2019. “The Effect of Cerebellar Repetitive Transcranial Magnetic Stimulation on Balance Function in Stroke Patients.” Medical Journal of Communications 33: 605–606.

[brb370471-bib-0069] Zhang, W. , L. Dai , W. Liu , et al. 2023. “The Effect and Optimal Parameters of Repetitive Transcranial Magnetic Stimulation on Lower Extremity Motor Function in Stroke Patient: A Systematic Review and Meta‐Analysis.” Disability and Rehabilitation 1–12. 10.1080/09638288.2023.2283605 37991330

[brb370471-bib-0070] Zhu, P. A. , Z. L. Li , Q. Q. Lu , et al. 2024. “Can Cerebellar Theta‐Burst Stimulation Improve Balance Function and Gait in Stroke Patients? A Randomized Controlled Trial.” European Journal of Physical Rehabilitation Medicine 60, no. 3: 391–399. 38577727 10.23736/S1973-9087.24.08307-2 PMC11255874

